# Peptide-based inhibitors targeting the PD-1/PD-L1 axis: potential immunotherapeutics for cancer

**DOI:** 10.1016/j.tranon.2024.101892

**Published:** 2024-02-14

**Authors:** Magdalena Bojko, Katarzyna Węgrzyn, Emilia Sikorska, Piotr Ciura, Claire Battin, Peter Steinberger, Katarzyna Magiera-Mularz, Grzegorz Dubin, Adam Kulesza, Adam K. Sieradzan, Marta Spodzieja, Sylwia Rodziewicz-Motowidło

**Affiliations:** a University of Gdańsk, Faculty of Chemistry, Wita Stwosza 63, 80-308 Gdańsk, Poland; b University of Gdańsk, Intercollegiate Faculty of Biotechnology of the University of Gdańsk and the Medical University of Gdańsk, Abrahama 58, 80-307 Gdańsk, Poland; c Medical University of Vienna, Institute of Immunology, Division of Immune Receptors and T cell Activation, Lazarettgasse 19, 1090 Vienna, Austria; d Małopolska Centre of Biotechnology, Jagiellonian University, Gronostajowa 7A, 30-387 Kraków, Poland; e Jagiellonian University, Faculty of Chemistry, Gronostajowa 2, 30-387 Kraków, Poland

**Keywords:** PD-1 - Programmed cell death 1, PD-L1 - Programmed cell death 1 - ligand 1, Immune checkpoint inhibitor, Disulfide-linked peptide, Immunotherapy, Cancer

## Abstract

The PD-1/PD-L1 complex belongs to the group of inhibitory immune checkpoints and plays a critical role in immune regulation. The PD-1/PD-L1 axis is also responsible for immune evasion of cancer cells, and this complex is one of the main targets of immunotherapies used in oncology. Treatment using immune checkpoint inhibitors is mainly based on antibodies. This approach has great therapeutic potential; however, it also has major drawbacks and can induce immune-related adverse events. Thus, there is a strong need for alternative, non-antibody-based therapies using small molecules, peptides, or peptidomimetics. In the present study, we designed, synthesized, and evaluated a set of PD-1-targeting peptides based on the sequence and structure of PD-L1. The binding of these peptides to PD-1 was investigated using SPR and ELISA. We also assessed their ability to compete with PD-L1 for binding to PD-1 and their inhibitory properties against the PD-1/PD-L1 complex at the cellular level. The best results were obtained for the peptide PD-L1(111–127)^(Y112C-I126C)^, named **(L11)**, which displaced PD-L1 from binding to PD-1 in the competitive assay and inhibited the formation of the PD-1/PD-L1 complex. The **(L11)** peptide also exhibited strong affinity for PD-1. NMR studies revealed that **(L11)** does not form a well-defined secondary structure; however, MD simulation indicated that **(L11)** binds to PD-1 at the same place as PD-L1. After further optimization of the structure, the peptide inhibitor obtained in this study could also be used as a potential therapeutic compound targeting the PD-1/PD-L1 axis.

## Highlights

• These studies concern blocking of the PD-1/PD-L1 complex formation.
• PD-L1 amino acid sequence was used to design complex inhibitors.
• The interaction of peptides with PD-1 was confirmed using SPR and ELISA.
• The peptides inhibit PD-1/PD-L1 binding in cellular assays.
• NMR and MD were used to confirm the peptide binding to PD-1.

## Graphical abstract

**Figure:**
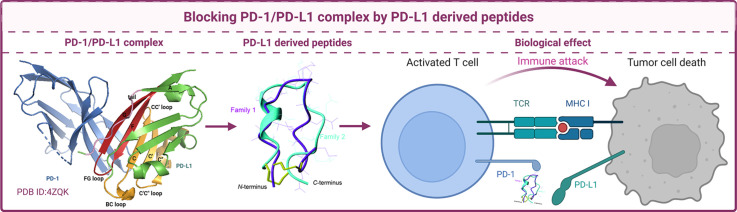

**Abbreviations**
**Ab**: antibody
**ANOVA**: analysis of variance
**APC**: antigen-presenting cell
**ATP**: adenosine triphosphate
**BTLA**: B- and T-lymphocyte attenuator
**DSS**: sodium 2,2-dimethyl-2-silapentane-5-sulfonate
**ELISA**: enzyme-linked immunosorbent assay
**eGFP**: enhanced green fluorescence protein
**Fc**: fragment crystallizable
**FBS**: foetal bovine serum
**FDA**: Food and Drug Administration
**gMFI**: geometric mean fluorescence intensity
**HRP**: horseradish peroxidase
**HVEM**: herpes virus entry mediator
**IgSF**: immunoglobulin superfamily
**irAE**: immune-related adverse events
**k_a_**: association constant
**k_d_**: dissociation constant
**K_D_**: equilibrium dissociation constant
**mAb**: monoclonal antibody
**mb aCD3**: membrane-bound anti-human CD3 single-chain variable fragment
**MD**: molecular dynamics
**MHC**: major histocompatibility complex
**MM/GBSA**: molecular mechanics-generalized Born surface area
**MREMD**: multiplexed-replica exchange molecular dynamics
**NMR**: nuclear magnetic resonance
**PD-1**: programmed cell death 1
**PD-L1**: programmed cell death 1 - ligand 1
**PE**: R-phycoerythrin
**PMI**: principal moment of inertia
**RMSD**: root-mean-square deviation
**RP-HPLC**: reversed phase-high performance liquid chromatography
**RPMI 1640**: Roswell Park Memorial Institute 1640 medium
**RU**: resonance unit
**SA**: simulated annealing
**scFv**: single-chain variable fragment
**SD**: standard deviation
**SPPS**: solid-phase peptide synthesis
**SPR**: surface plasmon resonance
**TBTU**: 2-(1H-benzotriazole-1-yl)−1,1,3,3 -tetramethylaminium tetrafluoroborate
**TCR**: T cell receptor
**TCS**: T cell stimulator
**TIL**: tumour-infiltrating lymphocyte
**TMB**: 3,3′,5,5′-tetramethylbenzidine
**UNRES**: UNited RESidue

## Introduction

Immunotherapy can be considered as a major breakthrough in cancer treatment. As confirmed in various experiments and clinical studies, this therapeutic approach significantly prolongs progression-free survival and overall survival of cancer patients [1], [2], [3]. Immunotherapy differs from other cancer treatment methods in that it does not directly target cancer cells, but instead stimulates the host immune system to fight them [4]. One such immunotherapeutic approach involves blocking the axis of inhibitory immune checkpoint proteins [5]. These proteins are often used by tumours as a mechanism to evade immune surveillance. One of the best known immune checkpoint protein complexes is the one formed by the receptor programmed cell death 1 (PD-1) and its ligand, namely programmed cell death 1 ligand 1 (PD-L1) [6]. Under physiological conditions, the interaction between PD-1 and PD-L1 is essential for the development of immune tolerance, which prevents excessive immune cell activity from causing tissue destruction and autoimmunity [7,8]. Studies on PD-1/PD-L1-mediated inhibition have shown that this inhibitory pathway is also an important component of the tumour immune evasion mechanism, which prevents the proliferation and differentiation of naïve T cells and contributes to T cell anergy and exhaustion [9,10]. Knowledge regarding exploitation of the PD-1/PD-L1 pathway by tumours and the possibility to influence this axis as a potential therapeutic target is rapidly expanding. In many types of cancers, PD-1 is highly expressed in tumour-infiltrating lymphocytes (TILs), including both CD4+ and CD8+ T cells, and the upregulated protein expression is correlated with tumour size and worse overall survival of cancer patients [11,12]. PD-L1 is commonly upregulated in cancer cells and in antigen-presenting cells in the tumour microenvironment, which greatly affects the regulation of T cell immunity [13], [14], [15], [16]. Many studies have highlighted that the inhibition of the PD-1/PD-L1 pathway is a very effective tumour treatment approach; this observation provides the scientific rationale for exploring immune checkpoint inhibitors for application in clinical oncology.

Targeting the PD-1/PD-L1 axis is a breakthrough in cancer treatment, and distinct types of anti-PD-1 and anti-PD-L1 antibodies have been used in cancer immunotherapy. In recent years, the Food and Drug Administration (FDA) has approved four anti-PD-1 monoclonal antibodies (mAbs), namely pembrolizumab, nivolumab, cemiplimab, and tislelizumab, and three anti-PD-L1 antibodies, namely atezolizumab, avelumab, and durvalumab, for treating several types of cancers [17]. These mAbs are used alone or in combination with other therapeutic agents for treating non-small cell lung cancer, head and neck squamous cell carcinoma, renal cell carcinoma, urothelial cancers, melanoma, classical Hodgkin's lymphoma, and many other cancers [18], [19], [20], [21]. However, this treatment produced successful, durable, and long-lasting responses only in a fraction of patients. Moreover, during treatment with mAbs, immune-related adverse events (irAEs) have been observed, and patient deaths have also been reported [22,23].

In 2015, the first structure of the human PD-1 and PD-L1 complex was obtained by Zak et al. [24]. Since then, the rational design of compounds blocking the PD-1/PD-L1 complex, other than antibodies, has gained momentum. In the last few years, peptides, peptidomimetics, and small molecules have been designed and tested as inhibitors of the PD-1/PD-L1 complex [25], [26], [27]. PD-1 and its ligand PD-L1 are transmembrane glycoproteins and belong to the immunoglobulin superfamily (IgSF). Both proteins contain a single IgV domain in their extracellular region; additionally, in PD-L1, a C-terminal IgC2-type domain has been identified [28,29]. The IgV domains of both proteins are similar and form two β-sheets with antiparallel βstrands, which are involved in the interaction between the proteins. The proteins bind in 1:1 stoichiometry. The total surface area of the protein-protein complex interface covers 1970 Å^2^ and involves the front faces of the βsheets of the IgV domains from both proteins (GFCC’ βsheets). Both polar and nonpolar interactions are involved in complex formation. Hydrophobic residues from the PD-1 and PD-L1 β-sheets form a core comprising V64, I126, L128, A132, and I134 residues and I54_L_, Y56_L_, M115_L_, A121_L_, and Y123_L_ residues, respectively (in this study, amino acid residues from PD-L1 are tagged with “_L_”). The hydrophobic core is surrounded by hydrophilic residues. Three structures focusing hot spots residues may be distinguished on the surface of the PD-L1 protein, namely one groove and two pockets - which interact with the appropriate residues in PD-1. The shallow groove contains D122_L_, Y123_L_, K124_L_, and R125_L_ from the C-terminal part of PD-L1 and D26_L_ from the N-terminal fragment of the protein. Three amino acid residues (Y68, Q75, and T76) from PD-1 are also located in this groove. The first pocket in PD-L1 consists of M115_L_, A121_L_, and Y123_L_ and accommodates I126 from PD-1, while the second pocket in PD-L1 consists of Y56_L_, E58_L_, R113_L_, M115_L_, and Y123_L_ and accommodates I134 from PD-1 [24].

In the present study, the crystal structure of the PD-1/PD-L1 protein complex (PDB: 4ZQK) [24] and molecular mechanics-generalized Born surface area (MM/GBSA) analysis, which was previously performed by us [30], were used to design peptides targeting PD-1. Three groups of compounds comprising three binding fragments of PD-L1 were synthesized and tested. The interactions of these PD-L1-derived peptides with PD-1 were confirmed by surface plasmon resonance (SPR) and enzyme-linked immunosorbent assay (ELISA). The ability of the peptides to compete with PD-L1 for binding to PD-1 and their inhibitory properties were studied by cellular assays. Moreover, for the peptide with the best inhibitory properties, namely PD-L1(111–127)^(Y112C-I126C)^ **(L11)**, the 3D structure was determined by nuclear magnetic resonance (NMR), and its interaction with PD-1 was confirmed by molecular dynamics (MD) methods. The obtained results indicate that PD-L1-derived peptides can be used as potential inhibitors of PD-1/PD-L1 complex formation; moreover, it is worth considering further modification of these peptides structure to improve their binding to the PD-1 protein and increase their inhibitory properties.

## Results

### Design and synthesis of PD-1/PD-L1 inhibitors targeting PD-1

In our previous studies, MM/GBSA analysis was performed using the crystal structure of the PD-1/PD-L1 complex (PDB: 4ZQK). This method allows to predict the binding free energy of interacting molecules and is commonly used for in silico characterization of receptor/ligand interactions [31]. The energy decomposition on per-residue and pairwise per-residue were considered to determine the amino acids crucial for binding of the proteins. According to the MM/GBSA analysis, three fragments of PD-L1 are robustly involved in the interaction with PD-1; this finding is in full agreement with the data obtained from the crystal structure of the proteins. The first fragment contains only one amino acid from the N-terminal part of PD-L1, specifically F19_L_ (−3.59 kcal/mol). The second fragment includes amino acid residues from the middle part of the protein, namely I54_L_ (−1.39 kcal/mol) and Y56_L_ (−2.74 kcal/mol). However, the amino acids with the lowest energy are mainly located in the C-terminal part of PD-L1; these include R113_L_ (−4.68 kcal/mol), M115_L_ (−2.35 kcal/mol), A121_L_ (−4.57 kcal/mol), D122_L_ (−1.75 kcal/mol), Y123_L_ (−3.30 kcal/mol), and R125_L_ (−6.31 kcal/mol) (Figure S1) [30].

The pairwise per-residue energy decomposition confirmed the strongest interactions between the amino acids from the C-terminal part of PD-L1 and PD-1. R113_L_ creates a strong interaction with E136 (−11.30 kcal/mol) and a weaker one with I134 (−1.84 kcal/mol). The second arginine residue important for complex formation is located at position 125 (R125_L_) in PD-L1; this residue interacts with E136 (−7.32 kcal/mol), additionally binds to Q75 (−5.77 kcal/mol), N74 (−2.61 kcal/mol), and T76 (−2.40 kcal/mol). The interactions between R123_L_ and R125_L_ with E136 are two of the strongest interactions between amino acids of PD-1 and PD-L1. A121_L_ forms a strong contact with K78 (−4.00 kcal/mol) and a moderate interaction with N66 (−1.86 kcal/mol). D122_L_ forms energetically important interactions with K78 (−5.25 kcal/mol) and Y68 (−5.20 kcal/mol), while it has a weaker one with N66 (−1.86 kcal/mol). Additionally, Y123_L_ interacts with E136 (−3.64 kcal/mol); however, this interaction is not as strong as that for R123_L_ and R125_L_. Moreover, Y123_L_ also interacts with I134 (−2.60 kcal/mol) and Y68 (−2.11 kcal/mol). The last essential amino acid for the PD-1/PD-L1 complex from this part of PD-L1 is K124_L,_ which binds to D77 (−6.38 kcal/mol) and T76 (−2.65 kcal/mol). The amino acids from the N-terminal and middle part of PD-L1 create notably fewer contacts with PD-1; the most important interactions are created by F19_L_ and K78 (−5.57 kcal/mol), D26_L_ and Q75 (−3.09 kcal/mol), Y56_L_ and A132 (−2.19 kcal/mol), and Q66_L_ and A132 (−1.95 kcal/mol). Moreover, A18_L_ strongly interacts with E84 (−9.80 kcal/mol), D85 (−2.06 kcal/mol), and R86 (−3.90 kcal/mol), while its per-residue energy decomposition is 3.46 kcal/mol; this finding indicates that it is unfavourable for protein complex formation [30]. The interactions between the individual amino acids in PD-L1 and PD-1 designated based on the crystal structure of the proteins and from pairwise per-residue energy decomposition are presented in Table S1. These data and the crystal structure of the protein complex enabled us to design three groups of peptides – potential PD-1 binders, which are shown in Table 1.

**Table 1:** Amino acid sequences and position in the protein of the designed peptides. X_1_ – norleucine, X_2_ – 2-aminobutyric acid; (&) – position of disulfide bond.

**No.**		**Peptide**	**Amino acid sequence**
**L1**	**Group I**	PD-L1(19–26)	Ac-FTVTVPKD-NH_2_
**L2**	**Group II**	PD-L1(52–68)	Ac-ALIVYWEX_1_EDKNIIQFV-NH_2_
**L3**		PD-L1(52–73)	Ac-ALIVYWEX_1_EDKNIIQFVHGEED-NH_2_
**L4**		PD-L1(52–79)	Ac-ALIVYWEX_1_EDKNIIQFVHGEEDLKVQHS-NH_2_
**L5**		PD-L1(45–68)	Ac-EKQLDLAALIVYWEX_1_EDKNIIQFV-NH_2_
**L6**		PD-L1(56–66)	Ac-YWEX_1_EDKNIIQ-NH_2_
**L7**	**Group III**	PD-L1(111–127)	Ac-VYRX_2_X_1_ISYGGADYKRIT-NH_2_
**L8**		PD-L1(113–126)	Ac-RX_2_X__1__ISYGGADYKRI-NH_2_
**L9**		PD-L1(113–126)^(C114-K124C)^	Ac-RC(&)X_1_ISYGGADYC(&)RI-NH_2_
**L10**		PD-L1(110–128)^(V111C-T127C)^	Ac-GC(&)YRX_2_X__1__ISYGGADYKRIC(&)V-NH_2_
**L11**		PD-L1(111–127)^(Y112C-I126C)^	Ac-VC(&)RX_2_X__1__ISYGGADYKRC(&)T-NH_2_
**L12**		PD-L1(113–126)^(C114-K124C)G120S^	Ac-RC(&)X_1_ISYGSADYC(&)RI-NH_2_
**L13**		PD-L1(113–126)^(C114-K124C)G120F^	Ac-RC(&)X_1_ISYGFADYC(&)RI-NH_2_
**L14**		PD-L1(113–126)^(C114-K124C)G120E^	Ac-RC(&)X_1_ISYGEADYC(&)RI-NH_2_
**L15**		PD-L1(121–125)	Ac-ADYKR-NH_2_

**Fig. 1:**
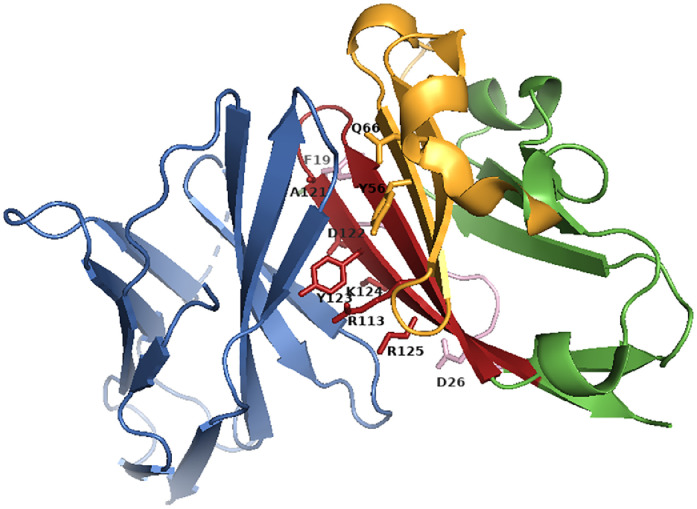
Structure of the PD-1/PD-L1 complex (PDB: 4ZQK) with the designed peptide groups marked on it; PD-1: blue, PD-L1: green. The PD-L1 fragments used to design three groups of peptides are marked as follows: purple - Group I, yellow - Group II, red - Group III. The amino acids in PD-L1 crucial for interaction with PD-1 are represented as sticks.

Group I (Table 1) contains only a single peptide, PD-L1(19–26) **(L1)**, which includes the amino acids from the N-terminal part of the IgV domain of PD-L1, ranging from F19_L_, which is crucial for protein binding, to D26_L_, which is a part of the shallow groove in the PD-L1 structure. Group II includes a series of peptides **(L2-L6)** from the middle region of the PD-L1 IgV domain. The shortest peptide **(L2)** contains two amino acids (I54_L_ and Y56_L_) important for the interaction of the proteins and was poorly soluble in water. To increase solubility, its sequence was elongated at the N- and C-terminals by adding hydrophilic amino acids from the protein sequence. The following peptides were thus obtained: PD-L1(52–73) **(L3)**, PD-L1(52–79) **(L4)**, PD-L1(45–68) **(L5)**, and PD-L1(56–66) **(L6)**. These alterations had no positive effect on the solubility of the peptides; therefore, Group II peptides (Table 1) were excluded from further analysis. Group III contains nine peptides **(L7-L15)** from the C-terminal part of the IgV domain of PD-L1. This group includes two linear peptides, namely PD-L1(111–127) **(L7)** and its shorter version PD-L1(113–126) **(L8)**. Both these peptides contain almost all the amino acid residues from the C-terminal part of the PD-L1 protein that are essential for interaction with PD-1 – specifically R113_L_, A121_L_, D122_L_, Y123_L_, K124_L_, and R125_L_ – except for M115_L_, which was substituted by norleucine (X_1_). Group III additionally includes six peptides stapled with intramolecular disulfide bonds; the first three of these peptides are PD-L1(113–126)^(C114-K124C)^ **(L9)**, PD-L1(110–128)^(V111C-T127C)^ **(L10)**, and PD-L1(111–127)^(Y112C-I126C)^ **(L11)**. Based on the results of MM/GBSA analysis, we also designed three analogues of peptide **(L9)** in which glycine 120 is replaced with serine, phenylalanine, or glutamic acid, namely PD-L1(113–126)^(C114-K124C)G120S^ **(L12)**, PD-L1(113–126)^(C114-K124C)G120F^ **(L13)**, and PD-L1(113–126)^(C114-K124C)G120E^ **(L14)**, respectively. The analysis of the structural features of the protein complex revealed that a cavity is formed near the G120 residue, which is energetically unfavourable. Certain amino acids were introduced to fill this cavity, as those residues can potentially form additional interactions with the nearby residues in PD-1. Group III is completed by the short peptide PD-L1(121–125) **(L15),** which contains five amino acids crucial for complex formation. The above-mentioned sequences correspond to the amino acid sequences of the PD-L1 protein in which the M59_L_ or M115_L_ residues have been substituted by norleucine (X_1_), while C114_L_, which creates a disulfide bond with C40_L,_ has been substituted by 2-aminobutyric acid (X_2_) in peptides **(L7), (L8), (L10)**, and **(L11)**. These modifications were introduced to prevent the oxidation of methionine and cysteine. The fragments in PD-L1 that were used to design the PD-L1-derived peptides are shown in Fig. 1, and the amino acids crucial for interaction with PD-1 are labelled.

All peptides were synthesized by solid-phase peptide synthesis (SPPS) technique and purified by reversed phase-high performance liquid chromatography (RP-HPLC). Some of these peptides were oxidized and subjected to another purification step.

### Evaluation of binding of PD-L1-derived peptides with PD-1 by ELISA and SPR

**Fig. 2:**
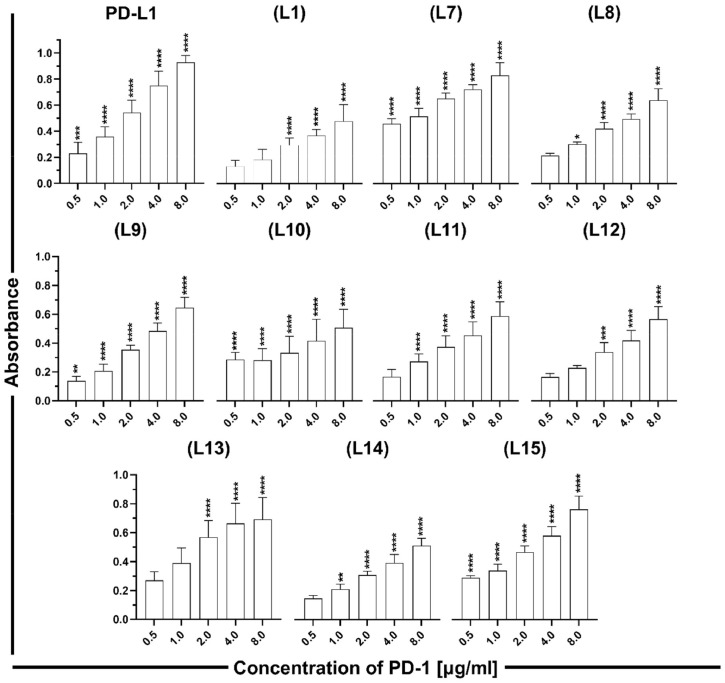
Binding of PD-1 to PD-L1 or to the indicated PD-L1-derived peptides analysed by indirect ELISA. Results are shown for at least three experiments performed independently in duplicate. Data are depicted as mean ± SD. Statistical analysis was performed using one-way analysis of variance (ANOVA) followed by Dunnet's post-hoc test. ****: *p* < 0.0001, ***: *p <* 0.001, **: *p <* 0.01, *: *p <* 0.05.

To evaluate the interaction of the designed peptides with the PD-1 protein, an ELISA was performed. The peptides, elongated at the N-terminal by a five-glycine linker and a biotin, were immobilized on the streptavidin plates. Subsequently, a fragment crystallizable (Fc) region fused with PD-1 was added at concentrations ranging from 8.00 to 0.5 µg/ml. The Fc region from PD-1 was used for recognition by anti-human IgG Ab conjugated with horseradish peroxidase (HRP). A colourless 3,3′,5,5′-tetramethylbenzidine (TMB) substrate was subsequently applied and converted by HRP to a blue reaction product, which absorbance was measured. PD-L1 was used as a positive control, and PBS-T was used as a negative control. The obtained results indicated that PD-1 binds to all the tested PD-L1-derived peptides in a concentration-dependent manner (Fig. 2). **(L7)** showed the strongest binding to PD-1 at all tested concentrations with statistical significance. Peptides **(L13)** and **(L15)** exhibited similar affinity to PD-1 as **(L7)**, particularly at higher concentrations. **(L15)** is the shortest peptide tested in this study, and it is the fragment of **(L7)** and **(L8). (L7)** and **(L8)** are linear peptides and cover the similar part of the PD-L1 sequence; however, compared to **(L8), (L7)** is elongated by two amino acids at the N-terminal and by one amino acid at the C-terminal. The protein binds to **(L7)** more strongly than to **(L8)**, although the additional amino acids are not crucial for protein binding. For the cyclic peptides, PD-L1 showed the strongest binding to **(L13)**. This peptide differs from **(L9), (L12)**, and **(L14)** by only one amino acid at position 120. **(L9)** binds to PD-L1 stronger than **(L12)** and **(L14)** and comparable to **(L11)**, which contains a disulfide bond at the different position. Peptides **(L1)** and **(L10)** showed the weakest interaction with the protein.

**Fig. 3:**
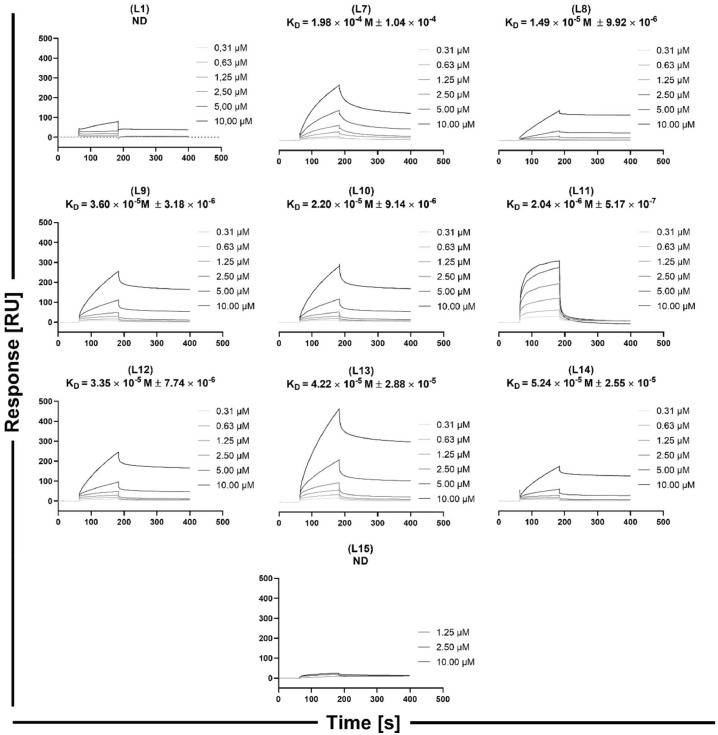
Sensorgrams of the PD-L1 derived peptides interacting with PD-1, obtained using the SPR technique. ND - not determined (no binding detected or binding too weak to establish reliable constants).

To obtain the kinetic parameters of the peptide/protein interaction the SPR technique was used. The tested peptides - elongated with a five-glycine linker and a biotin at the N-terminal - were immobilized on a streptavidin matrix-coated sensor chip (SA) and titrated with PD-1 at concentrations ranging from 0.31 to 10 µM. The interaction between PD-1 and biotinylated PD-L1 immobilized on the sensor was also investigated using the same parameters as those for peptide analysis. The equilibrium dissociation constant (K_D_) for the protein complex was estimated as 22.64 nM (Figure S2). In literature, the experimentally determined values of K_D_ are reported to range from 16 nM to 8.25 µM [32], [33], [34]; these constants slightly differ depending on the applied sensor chip (way of protein immobilization), protein (with or without glycans, or tag), and number of molecules immobilized on the sensor chip surface (mass transfer effect). The sensorgrams obtained for the peptides titrated with the PD-1 protein are shown in Fig. 3, and the binding kinetics parameters are presented in Table S2.

Peptide **(L1)** from group I and peptide **(L15)** from group III showed no interaction with PD-1 (Fig. 3), or the interaction was too weak to establish a reliable K_D_. The weakest quantifiable interaction was determined for the linear peptide **(L7)**, where the obtained K_D_ for the **(L7)**/PD-1 complex was 198 µM (Fig. 3, Table S2). The linear peptide **(L8)** exhibited stronger binding to PD-1 than **(L7)**; although the difference was approximately one order of magnitude, the obtained K_D_ was 14.9 µM. The analogues of peptide **(L8)**, with a disulfide bond at the same position, namely **(L9), (L12), (L13)**, and **(L14)**, showed comparable K_D_ values ranging from 52.4 to 33.5 µM, thus indicating that the substitution of G120_L_ with the other amino acids at this position does not contribute to the affinity. The longest peptide with a disulfide bond **(L10)** showed a similar K_D_ value to the one obtained for the aforementioned set of peptides, reaching 22.0 µM. Among all peptides, the strongest binding with PD-1 was observed for peptide **(L11)**, with a K_D_ value of 2.04 µM. **(L11)**/PD-1 showed a different kinetic profile than the other tested peptides from this group and was characterized by a fast association constant (k_a_) of 3.58 × 10^3^ M^−1^s^−1^, while the k_a_ values for the other peptides were in the range of 1.01 × 10^1^ – 3.30 × 10^1^ M^−1^s^−1^ (Table S2). **(L11)** showed faster dissociation than other peptides. The interaction profile of **(L11)** with PD-1 was similar to the one obtained for PD-1/PD-L1 (Figure S1).

According to the results of indirect ELISA, PD-1 interacts with all tested peptides. It should be noted that the peptide concentrations used in ELISA and SPR differed from each other; therefore, the binding strengths obtained with these two techniques cannot be compared. The results of ELISA also indicate that PD-1 binds to **(L1)** and **(L15)**. As previously mentioned, this interaction was not observed in the SPR analysis performed using the similar system where peptides were immobilized on the sensor chip. This difference might result from the number of molecules that bind to the surface (the plate for ELISA and the sensor chip for SPR) and accessibility of the peptides for the protein. Moreover, the incubation time of the peptides with the protein is apparently longer in ELISA than in SPR. First, the PD-1 protein might need more time to bind to the short, linear peptides immobilized on the sensor. Second, SPR measurement is performed in the continuous flow of the protein through the sensor surface, which might affect the binding of the protein to peptides. Considering these differences, these two experiments should be considered complementary to each other and not inconsistent.

### Evaluation of peptide behaviour – stability and cytotoxicity

After evaluation of the binding strength of the designed peptides to PD-1, their stability in the medium used in cell cultures and their effect on cell viability were assessed. The PD-L1-derived peptides were incubated with Roswell Park Memorial Institute 1640 (RPMI 1640) medium containing 10% of heat-inactivated foetal bovine serum (FBS). Samples were collected at 0 h and after 24 h of incubation and analysed by RP-HPLC. The peptide content in the samples was determined by comparing the corresponding peak areas to those of control samples, which constituted the corresponding peptides dissolved in H_2_O at 0 h. The conditions selected for the stability experiment were intended to simulate those used for cellular research.

**Fig. 4:**
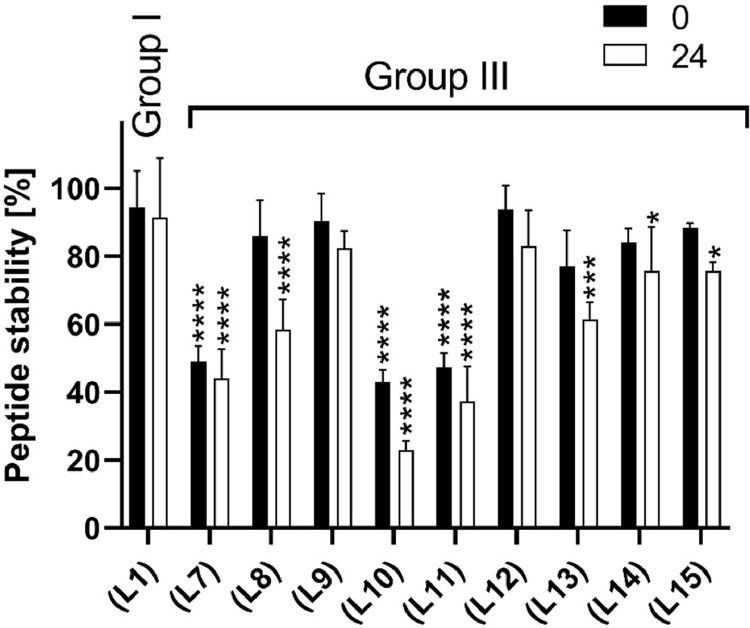
Peptide stability in RPMI 1640 medium at 0 and 24 h. The percentage of the remaining peptide was determined by comparing the peak area of control at 0 h with that for the collected samples. Concentrations were estimated by RP-HPLC. Results are shown for three experiments performed independently. Data are depicted as mean ± SD. Statistical analysis was performed using one-way ANOVA followed by Dunnet's post-hoc test. ****: *p <* 0.0001, *** *p <* 0.001, **: *p <* 0.01, *: *p <* 0.05.

The highest stability was observed for peptides **(L1), (L9)**, and **(L12)** (Fig. 4). The decrease in the amount of peptides in the samples was approximately 10 % at 0 h as compared to that in the controls. After 24 h of incubation, an additional, but only slight, decrease in the peptide content was noted (approximately 3 %, 8 %, and 11 %, respectively). Only minor changes were observed in the amount of peptides **(L13)** and **(L14)**. Before incubation, the amount of these peptides was 77 % and 84 %, while it was 61 % and 76 %, respectively, after 24 h of incubation. It should be noted that peptides **(L9)** and **(L12)** – **(L14)**, with the highest stability in the medium, cover the same fragment of PD-L1, have the same position of the disulfide bond, and differ only by one amino acid residue at position 120. For their linear analogue, peptide **(L8)**, a minor decrease in the concentration was observed, and its content was 86 % initially and 58 % after 24 h. The linear peptide **(L7)** was characterized by a significant drop of concentration to 49 % at 0 h and a further, though slight, decrease of 5 % was observed after 24 h of incubation. For its disulfide bond analogue, peptide **(L11)**, the amount of peptide before and after 24 h of incubation also drastically decreased, reaching 47 % and 37 %, respectively. Peptide **(L10)** exhibited a decrease in content for about 57 % at time 0 h and a further decrease for 20 % after 24 h of incubation. The last linear peptide **(L15)** displayed the least concentration fluctuation. At 0 h, it reached 88 % of the control concentration, and a further decrease of 12 % was observed after 24 h of incubation. The stability of the peptides in the medium is shown in Fig. 4, and the chromatograms registered for the selected peptides are shown in Fig. 3S. The decrease in the concentration of the peptides at 0 h is not the effect of their degradation, but it is probably because of the interaction of these peptides with medium components such as albumin contained in FBS. We previously observed similar effects for other peptides targeting the BTLA/HVEM axis [35], [36], [37]. These studies show that the peptides are present in the samples under the conditions in which cellular assays are conducted and have the potential to exert a biological effect.

**Fig. 5:**
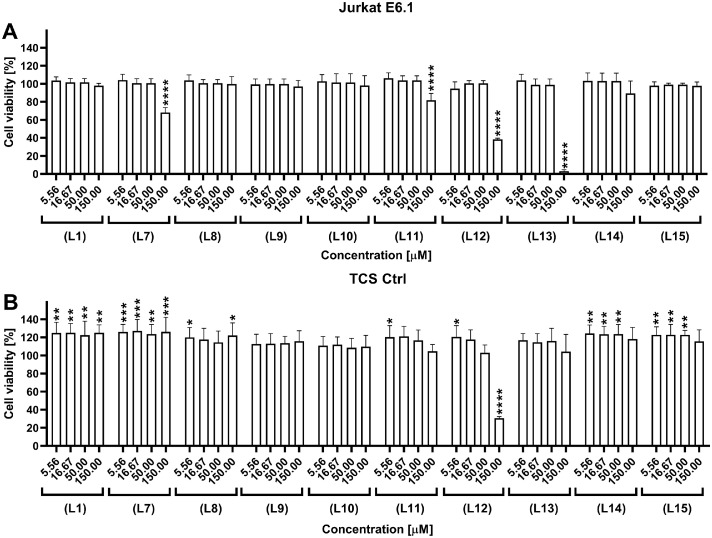
Cell viability assay used to determine the influence of the PD-L1-derived peptides on (A) Jurkat E6.1 cells and (B) TCS Ctrl cells lines after incubation for 24 h. Results are shown for three experiments performed independently in triplicate. Data are depicted as mean ± SD. Statistical analysis was performed using one-way ANOVA followed by Dunnet's post-hoc test. ****: *p <* 0.0001, ***: *p <* 0.001, **: *p <* 0.01, *: *p <* 0.05.

The influence of the peptides on Jurkat E6.1 cells and genetically modified BW5417 (T cell stimulators, TCS Ctrl) cells was subsequently examined. These two cell lines were used in the competition and inhibition bioassay described in the next paragraph. Cell viability was measured after 24 h of incubation with peptide concentrations ranging from 150.00 to 5.56 µM. The CellTiter-Glo® luminescence-based assay was used to estimate the number of viable cells. This test allowed determination of adenosine triphosphate (ATP), which is related to the metabolically active cells and proportional to the number of living cells . The results were compared and normalized to untreated cells (Fig. 5A). **(L12)** and **(L13)** peptides showed a significant negative influence on the number of Jurkat E6.1 cells at the highest tested concentration; which led to a decrease in the number of living cells to 38 % and 3 %, respectively. The peptides showed no negative effects at lower concentrations. Some decrease, though not significant, in cell viability was also observed for peptides **(L7)** and **(L11)** at the highest tested concentration, wherein the number of living, metabolically active cells decreased to 68 % and 82 %, respectively (Fig. 5A).

TCS Ctrl cells were less sensitive to the tested peptides than Jurkat E6.1 cells, and a significant decrease in cell viability was observed only for peptide **(L12)** at the highest tested concentration (Fig. 5B). However, an increase in cell viability (>20 %) was observed for the other tested peptides (Fig. 5B). The obtained results demonstrated that the tested peptides are not cytotoxic to the tested cells at concentrations below 50 µM, thus allowing to select suitable concentrations of peptides for cellular assays.

### Receptor-binding and competitive assay (displacing PD-L1 from the PD-1/PD-L1 complex)

A competition test was performed to determine the capacity of the PD-L1-derived peptides to inhibit the binding of PD-L1 to PD-1. PD-L1 with an Fc-tag and genetically modified Jurkat E6.1 cells with stable expression of PD-1 (JE6–1-NF-κB::eGFP PD-1) were used in this assay. The assay measured the binding of the ligand (PD-L1-Fc) to a target PD-1 protein located on modified Jurkat E6.1 cells in the presence of a second ligand (a PD-L1-derived peptide). The binding of the PD-L1-Fc protein was detected with a PE-labelled secondary anti-IgG Ab (Fc-specific). PD-1-expressing cells were incubated with peptides at three concentrations: 50.00, 16.67, and 5.56 μM, and PD-L1-Fc was used as a competitor. As a control, modified Jurkat E6.1 cells expressing PD-1 were incubated with the PD-L1-Fc protein in the absence of peptides. The results were analysed by flow cytometry.

**Fig. 6:**
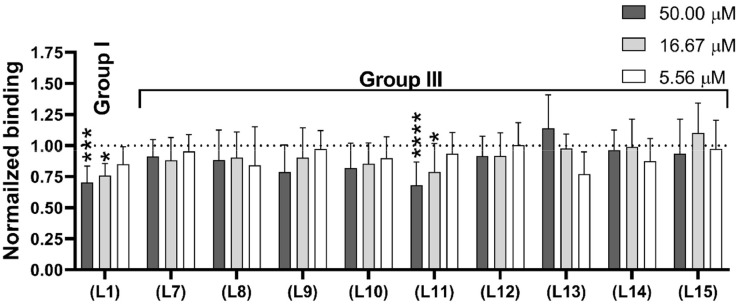
Competitive inhibition of PD-1/PD-L1 complex formation by the peptides obtained in this study. Modified Jurkat cells (JE6–1-NF-κB::eGFP PD-1) were incubated with the PD-L1-derived peptides, with the final concentration used in the experiment ranging from 50.00 to 5.56 µM. Subsequently, the cells were probed with PD-L1-Fc followed by PE-labelled anti-human IgG antibody and analysed by flow cytometry. The bar diagram shows the fold induction of geometric mean fluorescent intensity (gMFI) for at least three experiments performed independently in triplicate. Data were normalized to the gMFI obtained for the reporter PD-1 cell line treated with PD-L1-Fc in the absence of peptides. Data are depicted as mean ± SD. Statistical analysis was performed using one-way ANOVA followed by Dunnet's post-hoc test. ****: *p <* 0.0001, ***: *p <* 0.001, **: *p <* 0.01, *: *p <* 0.05.

The competitive assay showed that the best inhibitory properties were observed for peptides **(L1)** and **(L11)** in a concentration-dependent manner (Fig. 6). Slightly weaker blocking properties were also observed for peptides **(L9)** and **(L10)**, while no concentration-dependent effect was detected for the remaining tested peptides.

### Evaluation of PD-L1 peptides in a PD-1-T cell NF-κB-reporter system based assay

**Fig. 7:**
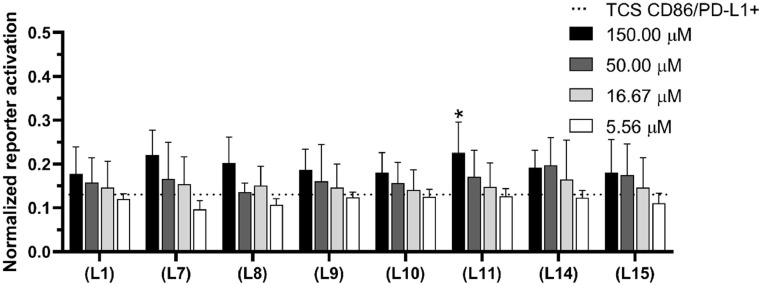
Inhibitory properties of the peptides in the functional cellular reporter assay. The PD-1 reporter cells were stimulated with TCS CD86/PD-L1 in the absence or presence of the peptides. The inhibitory properties of the peptides were measured based on eGFP expression by flow cytometry and normalized to gMFI eGFP obtained for the PD-1 reporter/TCS CD86 cells treated with the peptides. The dotted line shows the normalized eGFP expression level from the co-culture of the PD-1 reporter cells with TCS CD86/PD-L1. Results are shown for three experiments performed independently in duplicate. Data are depicted as mean ± SD. Statistical analysis was performed using one-way ANOVA followed by Dunnet's post-hoc test. ****: *p <* 0.0001, ***: *p <* 0.001, **: *p <* 0.01, *: *p <* 0.05.

To evaluate the capacity of the peptides to disrupt PD-1 and PD-L1 interaction, a cell co-culture system was used, which was established previously and comprised genetically modified Jurkat E6.1 and modified BW5147 cell lines [[38], [39], [40]]. The reporter assay used the following three cell lines: (**i**) a Jurkat E6.1 cell line expressing CD28 and PD-1, also known as the reporter cell line (JE6–1-NF-κB::eGFP PD-1), (**ii**) a control T cell stimulator (TCS CD86 Ctrl) constructed on the BW5147 cell line (PD-L1 negative, CD86 positive), and (**iii**) a T cell stimulator expressing PD-L1 (TCS CD86/PD-L1, PD-L1 positive, CD86 positive). The complete description of the cell lines used in the experiment is provided in Table S3. The reporter cell line shows endogenous expression of a TCR-CD3 complex, necessary for T cell activation, on its surface; it was lentivirally transduced to express response elements for nuclear factor-kappa light chain enhancer of activated B cells (NF-κB) that drives the expression of enhanced green fluorescent protein (eGFP) upon activation. The T cell stimulator cell lines TCS CD86 Ctrl and TCS CD86/PD-L1 express a membrane-bound anti-human CD3 single-chain variable fragment (scFv) (mb aCD3) that engages the CD3-TCR complex to yield the first signal required to stimulate T cells. Moreover, NF-κB-eGFP activation (signal 2) is enhanced by the interaction of CD86 on TCS with CD28 on modified Jurkat cells. The engagement of PD-L1 with PD-1 on the reporter cells results in NF-κB downregulation, thereby reducing eGFP expression. The inhibitory effects of the tested peptides on PD-1/PD-L1 complex formation were measured by the transcriptional level of eGFP (NF-κB::eGFP) through flow cytometry and compared with vehicle control; in particular, the eGFP expression level in the coculture of the PD-1 reporter cells with TCS CD86/PD-L1 was determined in the absence of peptides (Fig. 7, dotted line). The results were normalized to the geometric mean of fluorescence intensity (gMFI) of eGFP obtained for the PD-1 reporter/TCS CD86 cells (PD-L1-negative cells) treated with the selected peptides (Fig. 7). An anti-PD-1 mAb, namely pembrolizumab, was used as a positive control (Figure S4). The peptides were tested at concentrations between 150.00 and 5.56 µM.

All the tested peptides restored eGFP expression at the highest tested concentration (Fig. 7). A concentration-dependent effect was observed for peptides **(L1), (L7), (L9)**-**(L13)**, and **(L15)**; however, this effect was significant only for peptide **(L11)** at the highest tested concentration. Although peptides **(L12)** and **(L13)** restored eGFP expression at the highest tested concentration, they influenced eGFP expression by the reporter PD-1 cells stimulated with TCS CD86 Ctrl in the absence of PD-L1. This effect yielded a false positive result after normalization; hence, these data are not shown in Fig. 7 but are included in Figure S4. Contrary to peptides **(L12)** and **(L13)**, peptide **(L11)** showed no influence on eGFP expression by the reporter PD-1 cells stimulated with TCS CD86 Ctrl; hence, it can be considered as selective PD-1/PD-L1 inhibitor.

### Conformational studies for peptide PD-L1(111–127)^(Y112C-I126C)^ (L11)

Peptide **(L11)** demonstrated statistically significant inhibition of PD-1/PD-L1 complex formation. It competes with PD-L1 for binding to PD-1 and exhibits the strongest binding to PD-1 among all the tested PD-L1-derived peptides. NMR and MD simulation were subsequently performed to characterize the structure of this peptide. Assignments of ^1^H and ^13^C resonances were achieved based on homonuclear and heteronuclear 2D NMR spectra (Table S4 and Figure S5). The chemical shifts of ^13^C^β^ in cysteine residues were ∼ 42 ppm, which confirmed that thiol groups exist in the oxidized state [41]. The 94 (intraresidual and sequential) ^1^H–^1^H distance constraints evaluated from nuclear Overhauser effect spectroscopy (NOESY) were included in the protocol of simulated annealing (SA) to generate three-dimensional structures of the peptide. A set of 300 structures was obtained; subsequently, based on the cut-off value of 20 kcal/mol, 156 structures with the lowest energy value were selected from these 300 structures for further analysis. The selected conformations were clustered into 13 conformational families, two of which together accounted for 47 % of conformations, as shown in Figure S6. The results indicated high flexibility of the peptide and excluded formation of a well-defined secondary structure. The structures from both dominant conformational families showed a similar radius of gyration, specifically 8.31 ± 0.37 Å and 8.13 ± 0.34 Å. However, slight differences were noted in the shape of the structures from both families, based on the principal moment of inertia (PMI) analysis (Figure S7) [42]. In general, the structures of peptide **(L11)** were elongated; however, those from the second conformational family were closer to a sphere-like shape. The structure of the PD-L1(111–127) peptide fragment trimmed from the PD-1/PD-L1 complex and the structure of the two most important conformational families of peptide **(L11)** obtained from NMR are presented in Figure S8. It is apparent that the peptide structures obtained from NMR do not form a classic β-hairpin as the corresponding fragment of the protein.

**Fig. 8:**
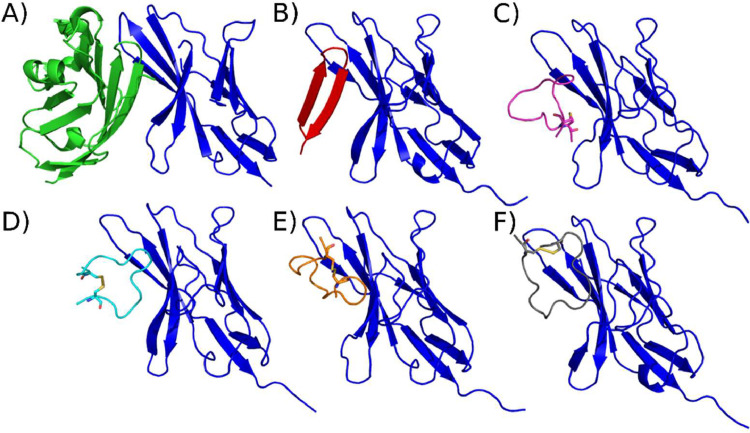
Structures A and B show, respectively, the complexes between PD-1 (blue) and PD-L1 (green), and between PD-1 (blue) and the PD-L1(111–127) fragment (red) trimmed from the PD-1/PD-L1 complex (PDB code: 4ZQK). Structures C-F show the complexes of peptide (**L11**) obtained from docking NMR structures to PD-1 by using the UNRES force field: (C) cluster from family 1 (lowest energy) – RMSD 3.84 Å, (D) cluster from family 1 (centroid) – RMSD 3.93 Å, (E) cluster from family 2 (lowest energy) – RMSD 4.86 Å, (F), cluster from family 2 (centroid) – RMSD 4.84 Å.

To determine the interface of PD-1 and **(L11)**, a molecular docking analysis was performed using a physics-based coarse-grained UNited RESidue (UNRES) force field [43,44]. Because the peptide structure exhibits high flexibility, docking simulations were performed for two conformational families obtained from NMR with two representatives from each family (the lowest energy and the family centroid). Our results showed that peptide **(L11)** bound to the PD-1 protein in the same place as the PD-L1 protein, regardless of the restraint set utilized (Fig. 8). For the first NMR family, the best root-mean-square deviation (RMSD) out of 10 clusters after docking was 3.84 and 3.93 Å for the restraints based on the lowest energy and the centroid, respectively, while the RMSD obtained for the second NMR family was 4.86 and 4.84 Å, respectively. The binding mode of the first NMR family of **(L11)** to PD-1 resembled the binding of the PD-L1 fragment used for peptide design in this study (Fig. 8A-D). For the structures based on the second NMR family, the peptide docked in a manner perpendicular to that observed for the PD-L1 fragment used for design it (Fig. 8E and F). The analysis of the interacting residues (Figure S9) revealed that lysine at position 14, norleucine (simulated as methionine) at position 5, and arginine at position 3 in peptide **(L11)** occur in most of the determined binding modes. These residues were also found to be in close contact with PD-1 in the crystal structure of the protein. Additionally, the amino acids I6, Y8, and G9 were involved in binding in the obtained modes; however, these residues were not found in close contact with PD-1 from the crystal structure. This difference can be explained by higher flexibility of the peptide and the ability to adjust its conformation.

## Discussion

As confirmed by many studies, T cells and effector molecules expressed by these cells are a crucial component in immune responses and play an essential role in anticancer activity [45]. T cell activation is controlled by two signals that are required to enable them to perform their effector functions. These signals are delivered when T cells interact with antigen‐presenting cells (APCs). T cell receptors (TCRs) are located on the surface of T cells, and these receptors are responsible for binding to peptides presented to them by a major histocompatibility complex (MHC) on the APC. This interaction delivers the first signal. The second signal is controlled by co-stimulatory and co-inhibitory molecules, also known as immune checkpoints. To induce the second signal, an immune checkpoint receptor, located on T cells, has to bind to its ligand on the APC [46,47]. Co-stimulatory and co-inhibitory immune checkpoints play critical roles in the maintenance of immune homeostasis, and they also participate in the immune response against many human diseases [47,48]. One of the best-known inhibitory immune checkpoints is the PD-1/PD-L1 complex. The binding of PD-1 on T cells to PD-L1 on tumour cells (which could mimic an APC) disrupts TCR signalling, thereby leading to the inhibition of T cell survival, proliferation and suppression of their effector function. Blocking the PD-1/PD-L1 pathway by the use of monoclonal antibodies has been shown to enhance antitumor T cell responses in many types of cancers [29,49]; however, there is still a need to search for compounds other than antibodies. Following the growing body of knowledge regarding the PD-1/PD-L1 complex interface, the number of alternative inhibitors to mAbs has increased. These include peptides and peptidomimetics, as shown in Tables S5 and S6 [[50], [51], [52], [53], [54], [55], [56], [57], [58], [59], [60]].

The crystal structure of the human PD-1/PD-L1 complex showed that the proteins bind in 1:1 stoichiometry; the total surface area of the protein-protein complex interface covers 1.970 Å^2^ and involves the front faces of the β-sheets of the IgV domains from both proteins (GFCCʹ β-sheets). Both polar and nonpolar interactions participate in complex formation. Hydrophobic residues from the PD-1 and PD-L1 β-sheets form a core comprising V64, I126, L128, A132, and I134 from PD-1 and I54_L_, Y56_L_, M115_L_, A121_L_, and Y123_L_ from PD-L1. The hydrophobic core is surrounded by hydrophilic residues. Three structures focusing hot spots residues occur on the surface of the PD-L1 protein - one groove and two pockets - which interact with the appropriate residues of PD-1. The shallow groove contains D122_L_, Y123_L_, K124_L_, and R125_L_ from the C-terminal part of PD-L1 and D26_L_ from the N-terminal fragment of the protein. Three amino acid residues (Y68, Q75, and T76) from PD-1 are also located in this groove. The first pocket in PD-L1 contains M115_L_, A121_L_, and Y123_L_ and accommodates I126 from PD-1, while the second pocket in PD-L1 contains Y56_L_, E58_L_, R113_L_, M115_L_, and Y123_L_ and accommodates I134 from PD-1 [24].

In our previous study, we reported the results of the MM/GBSA analysis, which enabled us to determine the amino acids crucial for protein binding. The MM/GBSA analysis for the PD-1/PD-L1 complex has also been performed by other researchers. The results of Huang et al. partially agree with our findings and highlight six hot spot residues for PD-L1: Y56_L_, Q66_L_, R113_L_, M115_L_, Y123_L_, and R125_L_. As noted by Huang et al., Q66_L_ forms a hydrogen bond with A132, and the energy of this interaction is -1.954 kcal/mol; the energy decomposition per-residue is only -0.296 kcal/mol, and it is therefore not marked here as a hot spot. These authors also confirm the importance of F19_L_, A121_L_, D122_L_, and I54_L_ [61]. The findings of Ding and Liu also agree with our results; however, these authors did not report F19_L_ as a critical amino acid for protein interaction [62].

In this study, based on the PD-L1 binding fragments, we designed 15 peptides to target PD-l. However, only 10 of these peptides were further evaluated, and the remaining were not tested due to their very poor solubility in water. All the examined peptides, except **(L1)**, cover the C-terminal fragment of PD-L1; which is a fragment of a two β-strands taking the form of a β-harpin structure. Disulfide bonds were introduced in some of the peptides in order to stabilize their structure. We did not staple peptide **(L1)** as it is a fragment of the flexible tail of PD-L1, and inducing a change in its structural organization could lead to the loss of affinity to PD-1. SPR and ELISA were performed to study the interaction of the PD-L1-derived peptides with PD-1. Peptide **(L11)** showed the strongest interaction with PD-1, with a K_D_ value of 2.04 µM for the complex. This peptide covers the C-terminal fragment of PD-L1 (amino acids 111 to 127) and contains R113_L_, A121_L_, D122_L_, Y123_L_, K124_L_, and R125_L_, which are crucial for proteins binding. This peptide possesses a disulfide bond, which was introduced by substitution of Y112_L_ and I126_L_ with cysteine residues and oxidation. Both these amino acids are not considered important for the interaction with PD-1. In comparison, the linear peptide covering the same fragment in PD-L1 – **(L7)** – interacts with PD-1 with a K_D_ value of 198 µM, which is approximately 97-fold weaker, thus providing the rationale for introducing a staple in **(L11)**. The obtained data agree with the results of Zhou et al. [54] and Abbas et al. [53]; this confirmed that cyclization and organization of the peptide structure can increase affinity to the β-sheet in proteins such as PD-1. Peptides containing the amino acid residues from G110_L_ to A132_L_ (DS-II), reported by Zhou et al., [54], and from Y112_L_ to T127_L_ (YT-16), reported by Abbas et al. [53], also possess disulfide bonds in their structure. Replacing the amino acid residues V111_L_ and T127_L_ with cysteine in peptide DSII and consequent creation of a disulfide bond increased the affinity to PD-1 by almost 4-fold in comparison to that for the linear peptide (K_D_ = 28 and 109 μM, respectively). Peptide **(L10)**, with a disulfide bond at the same position as DS-II, though shorter at the C-terminal by 4 amino acids, binds to PD-1 with a K_D_ value of 22.0 µM; this observation is comparable to the results of Zhou et al. This indicates that the last four residues are not essential for the interaction of the peptide with the target.

In peptide YT-16, reported by Abbas et al., C114_L_ – which occurs naturally in the PD-L1 sequence – and R125_L_, replaced by cysteine, were used to create a disulfide bond. Here, we tested peptides **(L8), (L9), (L12)** – **(L14)**, the first of which is linear, while the others possess a disulfide bond that was created using C114_L_ and residue K124_L_ substituted by cysteine. Peptide **(L9)** differs from YT-16 in that it lacks two amino acids, one each at the N- and C-terminals; moreover, in **(L9)**, K124_L_ rather than R125_L_ is substituted by cysteine. It is worth noting that both K124_L_ and R125_L_ are crucial for protein complex formation. The K_D_ values of YT-16 and **(L9)** were 17.8 nM and 36.0 µM, respectively. This finding indicates that K124_L_ is more important to create a strong interaction with PD-1 than R125_L_. On the other hand, this effect could also be due to the different spatial structural arrangement forced by the changing of the disulfide bond position in the amino acid sequence. Peptides **(L12), (L13)**, and **(L14)** additionally have G120_L_ replaced by phenylalanine, serine, or glutamic acid, respectively, in comparison to **(L9)**; which could form additional van der Waals interactions or be donors or acceptors of hydrogen bonds. All these compounds showed a significantly weaker interaction with the PD-l protein than YT-16, and their K_D_ values ranged from 14.9 to 52.4 µM. The C-terminal fragment of PD-L1 has been extensively researched, since the discovery of the PD-1/PD-L1 complex crystal structure [24]. Many linear, disulfide-bonded peptides and their analogues based on this fragment have been reported [55,58]. All these data indicate that slight changes in the amino acid sequence or disulfide bond position may affect the binding strength. We have previously investigated the importance of the position of the disulfide bond in the peptide amino acid sequence in studies focusing on the inhibitors of B- and T-lymphocyte attenuator and herpes virus entry mediator, and these prior studies reached the same conclusions on the importance of the position of stapling disulfide bonds [[35], [36], [37],^55^].

To better examine the potency of the PD-L1-derived peptides at the cellular level, competitive assays and stimulation assays were performed. Peptides **(L1)** and **(L11)** prevented PD-L1 binding in the competition assay in a concentration-dependent manner. **(L1)** did not show binding to PD-1 in SPR; however, it showed interaction with the protein in ELISA. These differences in obtained results may be due to the fact that in SPR and ELISA, the peptide is immobilized to the surface of the sensor or the plate, while it is in the solution in cellular assays. Moreover, the immobilization of the peptide may also lead to differences in its conformation as compared to that of the peptide in the solution. In the solution, a better fit to the PD-1 protein could be achieved, and the peptide could thus prevent PD-1/PD-L1 binding. Not only peptide **(L11)** displaces PD-L1 from the complex with PD-1, but all the PD-L1-derived peptides stimulate eGFP expression by inhibiting PD-1 signalling. **(L11)**, however, had the lowest stability value in the medium among all the tested peptides, thus suggesting that a structural modification aimed at increasing its stability is required for a better biological effect. Wang et al. reported peptides and peptidomimetics (linear, with a disulfide bond, tryptophan zipper, and D-proline in the peptide loop) comprising the amino acid residues from R113_L_ to I126_L_. One such peptide, namely P10.3, with D-proline in the loop and a tryptophan zipper in the positions S117W_L_ and D121W_L_, binds PD-1 with a K_D_ value of 1.80 μM and has the ability to restore IL-2 secretion in a co-culture model with activated Jurkat E6.1 and HCT-116 cells [56]. Boohaker et al. reported a linear peptide, namely PL120131, which consists of the amino acid residues from G120_L_ to D131_L_; this peptide reverses the apoptotic signal in murine primary lymphocytes and Jurkat cells induced by sPD-L1 [51]. For the peptides obtained in this study, similar investigations have not been conducted; therefore, their effect on T cells co-cultured with tumour cells requires further studies in the future.

Finally, the NMR structure of peptide **(L11)** was determined. **(L11)** does not have a well-defined structure; this indicates that the introduction of a disulfide bond in the amino acid sequence of the peptide is not sufficient to achieve the classical β-harpin structure. Nonetheless, according to the MD simulation, **(L11)** interacts with PD-1 at the same place as PD-L1, which explains some of its inhibitory properties. These findings indicate the importance of the C-terminal fragment in PD-L1 and show its potential as a starting point to design inhibitors for PD-1/PD-L1 complex formation. Moreover, the results demonstrate the need for further modifications of this fragment to improve its biological functions.

## Conclusion

Monoclonal antibodies are currently the state of the art in cancer immunotherapy targeting immune checkpoints; however, small molecules, peptides, and peptidomimetics have also shown the potential as future immunomodulatory drugs [63]. The findings of the present study and those reported by other authors [51,53,54,56] suggest that compounds covering the C-terminal fragment of the PD-L1 protein can recover immune cell functions compromised by PD-1/PD-L1 complex formation. Peptide **(L11)** binds to the PD-1 protein, competes with PD-L1 for binding to PD-1, and inhibits PD-1/PD-L1 formation. Moreover, the results of NMR analysis and MD simulation confirm the binding of this peptide to PD-1 at the same place as PD-L1, despite the fact that it does not have a similarly well-defined structure as the corresponding fragment in the protein. The obtained results strongly suggest that the structure of peptides has a crucial influence on their interaction with molecular targets, and slight changes in the amino acid sequence and structure of peptides can affect their inhibitory properties.

## Materials and methods

### Peptide synthesis and purification and formation of disulfide bonds

All PD-L1-derived peptides were synthesized by SPPS techniques using an automated microwave peptide synthesizer (Liberty Blue, CEM Corporation, Matthews, NC, USA). After synthesis, the N-terminal group of peptides was acetylated, or a biotin residue was coupled (the protocol is described below). The peptides were then cleaved from the resin and purified by RP-HPLC. Peptides with disulfide bonds were oxidized and purified again. All the applied procedures are reported in detail in our previous study [30].

### Biotin coupling to the N-terminal amine group of the peptide

The designed peptides were initially elongated at the N-terminal with a five-glycine linker using an automated microwave peptide synthesizer. The biotin residue was then coupled manually using 2.5 eq. of biotin (relative to resin loading), 2.36 eq. of 2-(1H-benzotriazole-1-yl)−1,1,3,3-tetramethylaminium tetrafluoroborate (TBTU) (relative to resin loading), and 2 eq. of *N,N*-diizopropylethylamine (DIPEA) (relative to biotin). Briefly, biotin and TBTU were dissolved in 3 ml of DMF, followed by the addition of DIPEA. After 3 min, the mixture was added to the peptidyl-resin, and the coupling reaction was run for 1 h with constant shaking. The mixture was then removed from the peptidyl-resin, and the procedure was repeated once with a new portion of the reagents. Finally, the peptidyl-resin was washed sequentially with DMF, DMF/DCM (1:1, v:v), and DCM. The remaining steps were the same as described previously [30].

### Indirect ELISA

ELISA was performed on 96-well streptavidin plates (Nunc, Thermo Fisher Scientific, #15124). Before the assay, the plates were prepared according to the manufacturer's instructions. The wells were then coated with 100 µl of biotinylated peptides or biotinylated PD-L1 (Sino Biological Company, China, #10084-H08H-B) at the concentration of 20 µg/ml and incubated for 1 h at 37°C with continuous shaking. After each step, the wells were washed (5 × 200 µl) with PBS-T (0.05 % Tween-20 in PBS with 0.3 M NaCl, pH 7.4). Subsequently, 2-fold serial dilutions of PD-1-Fc (Sino Biological Company, China, #10084-H08H-B) in PBS-T, at concentrations from 8.00 to 0.5 µg/ml, were added to the precoated wells. The mixture was then incubated for 2 h at 37°C with continuous shaking and washed five times. HRP-conjugated goat anti-human IgG Ab (Bio-Rad, #1721050) was used as the detection antibody at the concentration of 1:3000 (v:v). The plates were incubated with 100 µl of the detection antibody for 1 h at 37°C, with continuous shaking, and subsequently washed. In the last step, 100 µl of TMB (Thermo Scientific, #N301) was added to the wells for 15 min. After the incubation period, the absorbance was measured on an Infinite M200 Pro-plate reader (Tecan, USA), at the measurement wavelength of 650 nm and a reference wavelength of 492 nm. The assay was performed at least three times, and the results were analysed using GraphPad Prism 8 software.

### SPR analysis

Biacore T200 equipment (Cytiva, Marlborough, USA) was used to perform SPR analysis according to the manufacturer's protocol. PD-L1-derived peptides, elongated at the N-terminal with a five-glycine linker and a biotin, were suspended in PBS-P buffer (Cytiva, Marlborough, USA #28995084) and immobilized on the surface of an SA sensor chip (Cytiva, Marlborough, USA). The immobilization level was ∼800 resonance units (RU) for all peptides, except for **(L1),** whose final immobilization level was ∼500 RU. Different concentrations of PD-1 (Recepton Company, Poland, #R1–001–03) were prepared by a two-fold serial dilution method in PBS-P buffer to the values ranging from 10 to 0.31 µM and injected over the SA sensor chip with the immobilized peptides (titration of the PD-1 protein was performed at least three times). Biotinylated PD-L1 (Sino Biological Company, China, #10084-H08H-B) immobilized on an SA sensor chip was used as a control. All measurements were performed using PBS-P as a running buffer; the flow rate was set to 30 μl/min at 25°C. The chip was regenerated with 1.5 M NaCl and 10 mM glycine pH 3. The obtained data were analysed using Biacore T200 Evaluation Software (Cytiva, Marlborough, USA). The sensorgrams are presented for the results from which the background signal from the reference cell was subtracted.

### Peptide stability in the medium

Stability tests were performed using RPMI 1640 medium (Sigma-Aldrich, #R8758) with 10 % heat-inactivated FBS (Gibco, #10270–106) at pH 8.0. Samples were prepared by dissolving the peptides in H_2_O and adding them to the medium in the ratio of 1:3 (v:v). The final concentration of the peptides in the sample was 100 μM. The samples before analysis were collected at two time points: 0 h and 24 h of incubation (37°C with continuous stirring). The samples were then frozen in liquid N_2_ and stored at -80°C until the end of the experiment. Prior to analysis, the samples were thawed on ice, suspended in a 4-fold excess of absolute ethanol (v:v), and centrifuged (15,000 rpm, 4°C, 20 min). The supernatant was then transferred to test tubes, evaporated using a vacuum concentrator, then dissolved in 100 μl of 0.1 % trifluoroacetic acid (TFA) in H_2_O. The analysis was performed by analytical RP-HPLC using a Luna C18(2) (250 mm × 4.6 mm, 5 μm) column and a linear gradient from 5 % B to 100 % B in A over 60 min (A: 0.1 % TFA in H_2_O; B: 0.08 % TFA in 80 % ACN in H_2_O). Peptides that dissolved in H_2_O and the medium with 10 % FBS (without peptides) were used as controls.

### Cell lines

The Jurkat E6.1 cell line was purchased from Cell Line Service GmbH. The reporter cells constructed on the Jurkat E6.1 cell line and BW5147 cell lines (wild type and genetically modified – TCS cell lines) were derived from in-house stocks, generated, and cultured as described previously [38,40,64]. Jurkat E6.1 cells, reporter cells, BW5147 cells, and TCS cells were maintained in RPMI 1640 medium (Sigma-Aldrich, #R8758). All culture media were supplemented with 1 % penicillin and streptomycin (Sigma-Aldrich, #N0781) with the addition of 10 % heat-inactivated FBS (Gibco, #10270–106). The cell lines were incubated in a humidified incubator at 37°C under a 5 % CO_2_ atmosphere.

### Cell line viability assay

Cell line viability was examined on two cell lines – Jurkat E6.1 cells and TCS Ctrl cells. The experiments were performed on 96-well solid white plates for cell culture. Jurkat E6.1 cells and TCS Ctrl cells were seeded into wells on the day of the experiment at the density of 2 × 10^4^ cells per well in 50 µl RPMI 1640 medium containing 10 % FBS. Subsequently, 50 µl of peptides at 150 to 1.90 µM concentrations were added to the wells. Cell viability was measured using 100 µl of Cell Titter-Glo (Promega Corporation, #G7570) added to the wells. Luminescence was measured on a Spark M10 plate reader (Tecan, Switzerland) with an integration time of 0.5 s. The experiment was performed at least three times, and the results were analysed by GraphPad Prism 8 software. All the applied procedures are described in our previous study [30].

### Competitive assay at the cellular level

The competitive potency of the PD-L1 derived peptides was tested using the JE6–1-NF-κB::eGFP cell line expressing PD-1. The peptides were prepared by dissolving in H_2_O and diluted to the final concentration of 12 % of H_2_O by using PBS with 0.5 % FBS. Serial dilutions of the peptides were prepared in concentrations ranging from 50.00 to 5.56 μM. The experiment was conducted in 5 ml conical bottom tubes for flow cytometry. The peptides were incubated at the indicated concentrations with JE6–1-NF-κB::eGFP PD-1 cells for 120 min at 4°C. Subsequently, as a peptide competitor, the human PD-L1-Fc protein was added at the concentration of 1 μg/ml and incubated for 15 min at 4°C. Binding of PD-L1-Fc was detected by PE-labelled donkey anti-human IgG antibody (Jackson ImmunoResearch Europe Ltd., #709–116–098), which was incubated with each sample at the concentration of 1:300 (v:v) in PBS containing 0.5 % FBS for 20 min at 4°C. Each step of the experiment was followed by spinning (5 min, 500 rpm, 4°C) and washing of the samples with PBS containing 0.5 % FBS. The samples were analysed by flow cytometry, and the mean and standard deviation of gMFI of the viable cell population were determined. The experiment was performed at least three times in triplicate. Flow cytometry analysis was performed on a FACSCalibur flow cytometer (Becton Dickinson Immunocytometry System, USA) by using CellQuest software. Data were analysed with FlowJo (version 10.0.6, Tree Star, Ashland, OR, USA) and GraphPad Prism (version 8, GraphPad Software, Inc., La Jolla, CA, USA).

### Peptide inhibitory properties against the PD-1/PD-L1 axis

The experiment was performed on 96-well tissue culture-treated transparent plates by using RPMI 1640 medium containing 10 % FBS. The cells were seeded into the wells at the density of 5 × 10^4^ cells/well for reporter cells and 2 × 10^4^ cells/well for TCS cell lines. The peptides were prepared in three-fold serial dilutions in concentrations ranging from 150.00 to 5.56 μM and were preincubated with the reporter cells for 90 min. Subsequently, cells of the second cell line were added; co-culture was performed for 24 h at 37°C under a 5 % CO_2_ atmosphere_._ The peptides were dissolved in H_2_O and diluted to the desired final concentration in RPMI 1640 medium containing 10 % FBS, wherein the H_2_O concentration did not exceed 12 %. Pembrolizumab anti-PD-1 mAb (Keytruda®, MSD Sharp & Dohme GmbH) was used as a positive control. After 24 h, the cells were harvested, stained with APC-conjugated mCD45.2 mAb to exclude TCS from the analysis, and eGFP expression was measured by flow cytometry. The mean and standard deviation values of the gMFI of the viable cell population were determined. The experiment was performed at least three times in duplicate. To assess the surface expression of the receptors, the following antibodies from Biolegend (USA) were used in the flow cytometry analysis: allophycocyanin (APC)-conjugated anti-human PD-1 Ab (#EH12.2H7); PE-conjugated anti-human PD-L1 Ab (#29E.2A3); APC-conjugated anti-human CD86 Ab (#IT2.2); PE-Cy7-conjugated anti-human CD3 Ab (#UCHT-1); APC-conjugated anti-mouse CD45.2 Ab (#104); and APC-conjugated CD14 Ab (HCD14). The flow cytometry analysis was performed on a FACSCalibur flow cytometer (Becton Dickinson Immunocytometry System, USA) by using CellQuest software. Data were analysed with FlowJo (version 10.0.6, Tree Star, Ashland, OR) and GraphPad Prism (version 8, GraphPad Software, Inc., La Jolla, CA).

### NMR measurements

NMR experiments were conducted at 298 K on a Bruker Avance III 700 MHz (1^H^ frequency: 700.55 MHz) (Faculty of Chemistry, University of Gdańsk, Gdańsk, Poland) instrument operated at the magnetic field of 16.4 T. The initial concentration of the sample was 3.7 mM in H_2_O/D_2_O (v/v: 9:1). The following NMR experiments were conducted: 1D ^1^H, 80 ms TOCSY, 300 ms ROESY, 200 ms NOESY, and ^1^H–^13^C HSQC. The 2D heteronuclear spectrum was recorded according to the natural abundance of the ^13^C isotope. All chemical shifts were referenced with respect to the external compound sodium 2,2-dimethyl-2-silapentane-5-sulphonate (DSS) using Ξ = 0.251449530 ratio for indirectly referenced ^13^C resonances [65].

### Molecular modelling

MD simulations were performed with the ff14SB force field in the AMBER 16 package [66]. The structures of the peptides were computed using a SA algorithm with interproton distance restraints. The inter-proton distances were calculated on the basis of NOE intensities by the CALIBA algorithm of the CYANA 2.1 program [67]. SA started from a random conformation. A total of 300 SA cycles were performed where the *n*^th^ conformation initiated the *n* *+* *1* cycle. Each SA cycle comprised 30,000 MD steps (30 ps). The system was heated for 1 ps from 0 to 1200 K, equilibrated at 1200 K for 2 ps, and then cooled back to 298 K over 27 ps. During the cooling process, weak temperature coupling (slow cooling) was applied for the first 17 ps followed by strong temperature coupling (rapid cooling) for the last 10 ps. NMR interproton distance restraints were enforced during the annealing process with an increasing force constant from 25 to 50 kcal/(mol × Å^2^) over the first 3 ps and was maintained at 50 kcal/(mol × Å^2^) for the remaining calculations. The geometry of the peptide groups (all *trans*) was kept fixed according to the NMR data (*f* = 50 kcal/(mol × rad^2^)). To approximate solvent interaction, a generalized Born model was used [68]. Finally, the structures were minimized, and the conformations within a relative energy cut-off of 20 kcal/mol, with respect to the lowest energy conformation, were selected for further analysis. Visualization of the structures was achieved using the VMD program [69]. Moments of inertia were calculated using GROMACS 2019 tools [70].

### Molecular docking of (L11) peptide to PD-1 using an UNRES force field

To study the binding of peptide (**L11**) to PD-1 a physics-based coarse-grained UNRES force field was used [43,44]. Because the UNRES force field is available only for coded amino acids, all non-coded amino acids were substituted with their natural counterparts. Simulations were performed for two NMR families. As the peptide exhibits large conformational flexibility, the restraints were based on two structures for each family: specifically those based on conformations with the lowest energy and the structure closest to the NMR family centroid. The peptide was randomly rotated and placed in the proximity of the PD-1 protein, with the condition that the centres of mass of the peptide between placements could not be too close to each other (a variable distance criterion was used, which was gradually decreased if no satisfactory position was found over several attempts). Thus, as many different initial orientations as possible were obtained. The algorithm also generated files with the restraints, which retained the conformation of each chain within an RMSD of 2–3 Å and did not impact inter-chain orientations during subsequent coarse-grained simulations, as reported in our previous study [36]. No restraints were imposed on the PD-1-peptide interface. For each system (type of restraints), the multiplexed-replica exchange molecular dynamics (MREMD) docking simulation started from energy minimization of the generated orientations of molecules, which were subsequently subjected to 10 million MD steps per replica. The time step was set to 4.89 fs of UNRES time, and periodic boundary conditions with an implicit solvent were used. Twenty replicas in the temperature range of 250–400 K were run, with two replicas per temperature. The total simulation time reached 48.9 ns of UNRES time per replica, which equates to approximately 50 µs of real-time per replica due to smoothing of the energy landscape in the coarse-grained representation. Replica exchanges were attempted every 10,000 steps, and snapshots were saved with the same frequency. The box size was set to 300 × 300 × 300 Å. After simulation, a binless weighted histogram analysis method was used to obtain ensemble average properties at the given temperature. Clustering was then performed at 340 K to obtain representative structures at the given temperature. Ten most energetically favourable clusters were obtained using the Wards minimum variance algorithm. Subsequently, the binding modes were selected from the obtained 10 clusters based on the similarity to the original binding mode (according to the RMSD criterion). The contacts between PD-1 and **(L11)** were determined and visualized with LigPlus software [71].

## CRediT authorship contribution statement

**Magdalena Bojko:** Formal analysis, Investigation, Methodology, Validation, Visualization, Writing – review & editing, Writing – original draft. **Katarzyna Węgrzyn:** Formal analysis, Investigation, Methodology, Validation. **Emilia Sikorska:** Formal analysis, Investigation, Methodology, Software, Validation, Visualization. **Piotr Ciura:** Investigation. **Claire Battin:** Investigation, Methodology. **Peter Steinberger:** Investigation, Methodology. **Katarzyna Magiera-Mularz:** Investigation. **Grzegorz Dubin:** Funding acquisition, Investigation. **Adam Kulesza:** Investigation. **Adam K. Sieradzan:** Methodology, Software, Validation, Formal analysis, Investigation, Visualization. **Marta Spodzieja:** Conceptualization, Investigation, Methodology, Supervision, Writing – original draft, Writing – review & editing. **Sylwia Rodziewicz-Motowidło:** Conceptualization, Funding acquisition, Project administration, Supervision.

## Declaration of competing interest

The authors declare that they have no known competing financial interests or personal relationships that could have appeared to influence the work reported in this paper.

## Appendix Supplementary materials

Image, application 1
