# Supplementary material for: Peptide-based inhibitors targeting the PD-1/PD-L1 axis: potential immunotherapeutics for cancer
Source: Transl Oncol. 2024 Feb 14;42:101892. doi: 10.1016/j.tranon.2024.101892 (PMC10877416; doi:10.1016/j.tranon.2024.101892)
Supplement: Supplementary file 1 [file mmc1.docx]

**Peptide-Based Inhibitors Targeting the PD-1/PD-L1 Axis: Potential Immunotherapeutics for Cancer**

Magdalena Bojko^1^, Katarzyna Węgrzyn^2^, Emilia Sikorska^1^, Piotr Ciura^1^, Claire Battin^3^, Peter Steinberger^3^, Katarzyna Magiera-Mularz^4^, Grzegorz Dubin^5^, Adam Kulesza^1^, Adam K. Sieradzan^1^, Marta Spodzieja^1*^ and Sylwia Rodziewicz-Motowidło^1*^

^1^ University of Gdańsk, Faculty of Chemistry, Wita Stwosza 63, 80-308 Gdańsk, Poland

^2^ University of Gdańsk, Intercollegiate Faculty of Biotechnology of the University of Gdańsk and the Medical University of Gdańsk, Abrahama 58, 80-307 Gdańsk, Poland

^3^ Medical University of Vienna, Institute of Immunology, Division of Immune Receptors and T cell Activation, Lazarettgasse 19, 1090 Vienna, Austria

^4^ Małopolska Centre of Biotechnology, Jagiellonian University, Gronostajowa 7A, 30-387 Kraków, Poland

^5^ Jagiellonian University, Faculty of Chemistry, Gronostajowa 2, 30-387 Kraków, Poland

*Corresponding authors: Marta Spodzieja, [marta.spodzieja@ug.edu.pl](mailto:marta.spodzieja@ug.edu.pl); Sylwia Rodziewicz-Motowidło, [s.rodziewicz-motowidlo@ug.edu.pl](mailto:s.rodziewicz-motowidlo@ug.edu.pl)

*
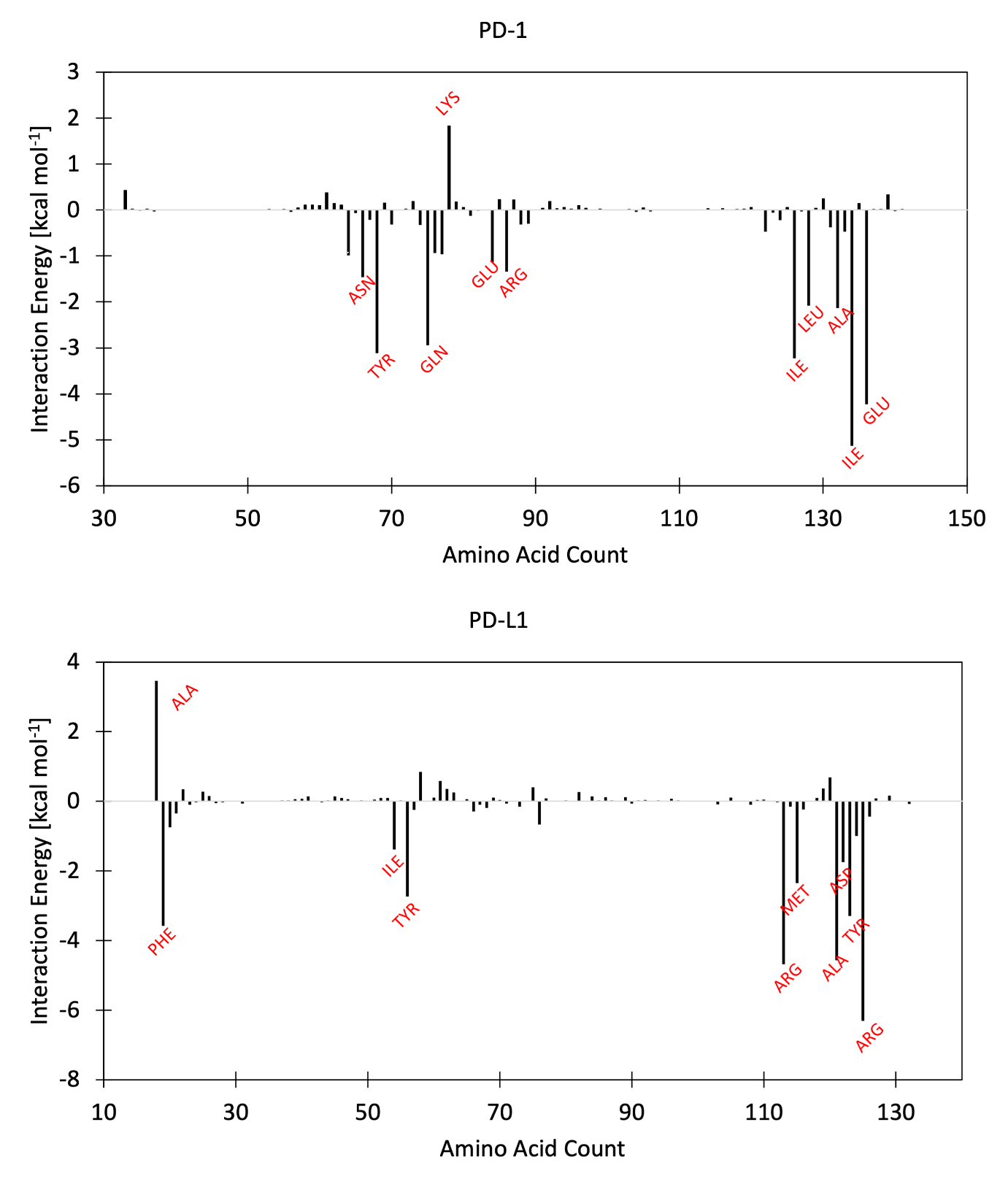
****Figure S1.*** *Energy decomposition on a per-residue basis for PD-L1 amino acid residues. To rank the interaction energy as “strong”, the* Δ*G value should be the least or lower than -1.0 kcal/mol* [1] *.*

***Table S1.*** *Amino acid residues and types of interaction responsible for PD-1/PD-L1 complex formation based on the crystal structure of PD-1/PD-L1 and the results of the MM/GBSA analysis. The amino acid residues important for the interaction were divided into three groups to reflect in which groups of the designed peptides they are present.*

| GROUP OF PEPTIDE | PD-L1 | | PD-1 | | Data from the crystal structure[2] | | Data from MM/GBSA analysis [1] |
| --- | --- | --- | --- | --- | --- | --- | --- |
|  | RName | RNum | RName | RNum | Type of interaction | Groups taking part in interaction | Interaction energy [kcal/mol] |
| I | PHE | 19 | LYS | 78 | H bond | amine hydrogen from side chain with carbonyl oxygen from main chain | -5.572 |
|  | ASP | 26 | GLN | 75 | H bond | amid hydrogen from side chain with carboxyl oxygen from side chain | -3.090 |
| II | TYR | 56 | ALA | 132 |  |  | -2.185 |
|  | GLN | 66 | ALA | 132 | H bond | amid hydrogen from side chain with carbonyl oxygen from main chain | -1.954 |
| III | ARG | 113 | GLU | 136 | Salt bridge | side chains | -11.303 |
|  | ARG | 113 | ILE | 134 |  |  | -1.844 |
|  | ALA | 121 | LYS | 78 |  |  | -3.996 |
|  | ALA | 121 | ASN | 66 | H bond | amid hydrogen from side chain with carbonyl oxygen from main chain | -1.860 |
|  | ASP | 122 | LYS | 78 |  |  | -5.245 |
|  | ASP | 122 | TYR | 68 | H bond | hydroxyl hydrogen from side chain with carboxyl oxygen from side chain | -5.200 |
|  | ASP | 122 | ASN | 66 |  |  | -1.864 |
|  | TYR | 123 | GLU | 136 | H bond | carboxyl oxygen from side chain with hydroxyl hydrogen from side chain | -3.642 |
|  | TYR | 123 | ILE | 134 | Alkyl- π | side chains | -2.600 |
|  | TYR | 123 | TYR | 68 | π - π | side chains | -2.107 |
|  | LYS | 124 | ASP | 77 |  |  | -6.381 |
|  | LYS | 124 | THR | 76 | H bond | carbonyl oxygen from main chain with amine hydrogen from side chine | -2.652 |
|  | ARG | 125 | GLU | 136 |  |  | -7.319 |
|  | ARG | 125 | GLN | 75 | H bond | carbonyl oxygen from side chain with amid hydrogen from main chain | -5.768 |
|  | ARG | 125 | ASN | 74 |  |  | -2.609 |
|  | ARG | 125 | THR | 76 |  |  | -2.396 |


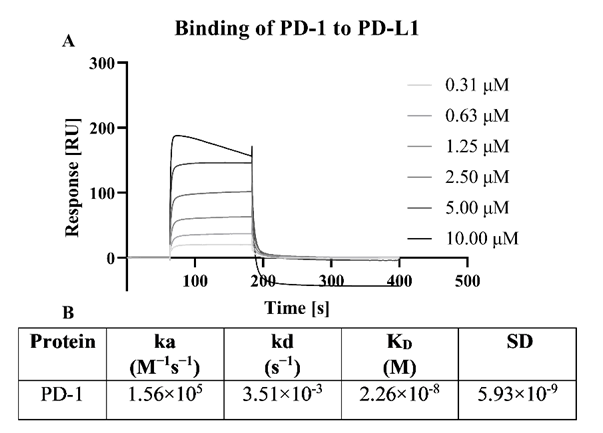


***Figure S2.*** *The SPR profile of PD-L1 treated by different concentrations of PD-1.
A) Sensorgrams showing the relation between the detected signal of PD-1 binding to PD-L1 in time. B) The binding kinetics parameters from the SPR analysis calculated with the Biacore T200 Evaluation Software.*

***Table S2.*** *Association and dissociation rates and equilibrium dissociation constants calculated for PD-1 binding to the PD-L1 derived peptides from the SPR data. Results were calculated using Biacore T200 Evaluation Software, from at least three independent titration analyses. The 1:1 binding model was applied. ND - not determined (no binding detected or binding too weak to establish reliable constants), SD – standard deviation.*

|  | **No** | **Peptides** | **k_a_**  **(M^-1^s^-1^)** | **k_d_**  **(s^-1^)** | **K_D_**  **(M)** | **SD** |
| --- | --- | --- | --- | --- | --- | --- |
| **Group I** | **L1** | PD-L1(19-26) | ND | ND | ND | ND |
| **Group III** | **L7** | PD-L1(111-127) | 1.09×10^1^ | 1.79×10^-3^ | 1.98×10^-4^ | 1.04×10^-4^ |
|  | **L8** | PD-L1(113-126) | 2.47×10^1^ | 1.94×10^-4^ | 1.49×10^-5^ | 9.92×10^-6^ |
|  | **L9** | PD-L1(113-126)^(C114-K124C)^ | 1.06×10^1^ | 3.99×10^-4^ | 3.60×10^-5^ | 3.18×10^-6^ |
|  | **L10** | PD-L1(110-128)^(V111C-T127C)^ | 3.31×10^1^ | 7.10×10^-4^ | 2.20×10^-5^ | 9.14×10^-6^ |
|  | **L11** | PD-L1(111-127)^(Y112C-I126C)^ | 3.58×10^3^ | 6.70×10^-3^ | 2.04×10^-6^ | 5.17×10^-7^ |
|  | **L12** | PD-L1(113-126)^(C114-K124C)G120S^ | 1.37×10^1^ | 4.49×10^-4^ | 3.35×10^-5^ | 7.74×10^-6^ |
|  | **L13** | PD-L1(113-126)^(C114-K124C)G120F^ | 1.94×10^1^ | 6.97×10^-4^ | 4.22×10^-5^ | 2.88×10^-5^ |
|  | **L14** | PD-L1(113-126)^(C114-K124C)G120E^ | 1.05×10^1^ | 4.89×10^-4^ | 5.24×10^-5^ | 2.55×10^-5^ |
|  | **L15** | PD-L1(121-125) | ND | ND | ND | ND |

| **(L15)** PD-L1(121-125) | **(L7)** PD-L1(111-127) |
| --- | --- |
| 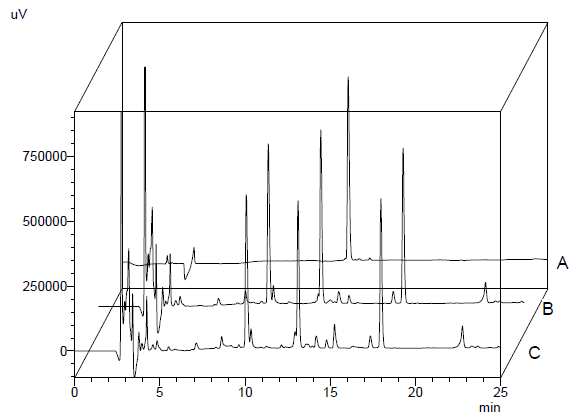 | 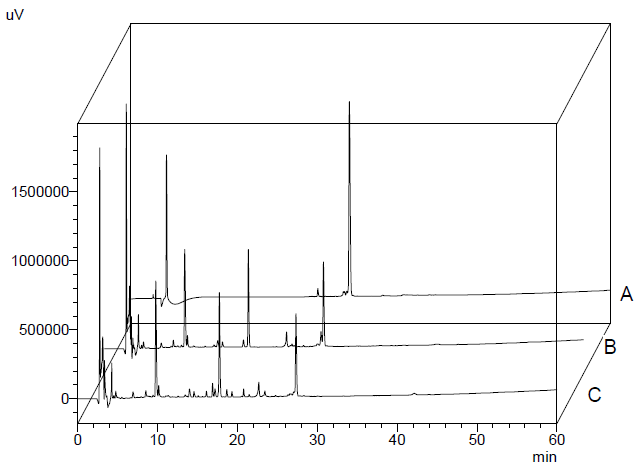 |
| **(L10)** PD-L1(110-128)^(V111C-T127C)^ | **(L11)** PD-L1(111-127)^(Y112C-I126C)^ |
| 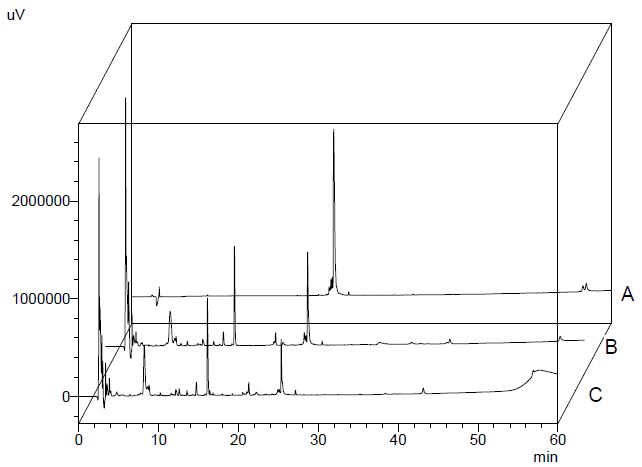 | 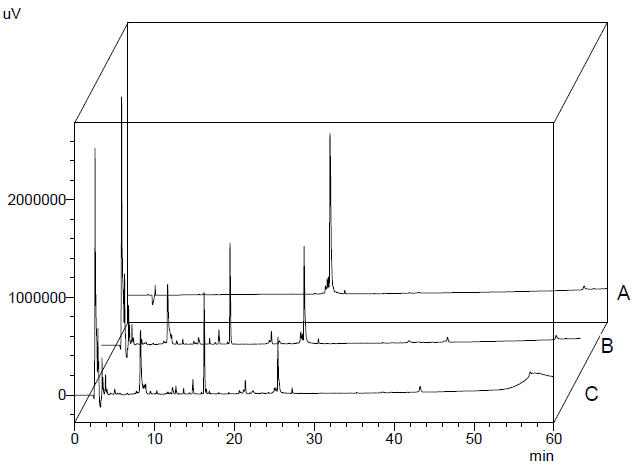 |

**Figure S3.** Chromatograms registered for peptides **(L15)**, **(L7)**, **(L10),** and **(L11)**. A) peptide in water t=0 h, B) peptide in medium t=0 h, C) peptide in medium t=24 h.

**Table S3.** Description of the cell lines used in the stimulation assay.

| **Cell line name** | **Description of the cell line** |
| --- | --- |
| **JE6-1-NF-κB::eGFP** | the NF-κB::eGFP reporter cells constructed on the human Jurkat E6.1 cell line expressing CD28 (without expression of PD-1), also known as the reporter Ctrl |
| **JE6-1-NF-κB::eGFP** **PD-1** | the NF-κB::eGFP reporter cells constructed on the human Jurkat E6.1 cell line expressing CD28 and PD-1, also known as the reporter PD-1 |
| **TCS CD86 Ctrl** | the cell line which express mb aCD3 and CD86 (CD86 interacts with CD28 on the J-NF-κB::eGFP cell line) constructed on the BW5147 cell line |
| **TCS CD86/PD-L1** | the cell line which express mb aCD3, CD86 and PD-L1 constructed on the BW5147 cell line |
| **wtBW** | wild type of BW5147 cell line – mb aCD3, CD86 and PD-L1 negative. |

| **A** | **B** |
| --- | --- |
| **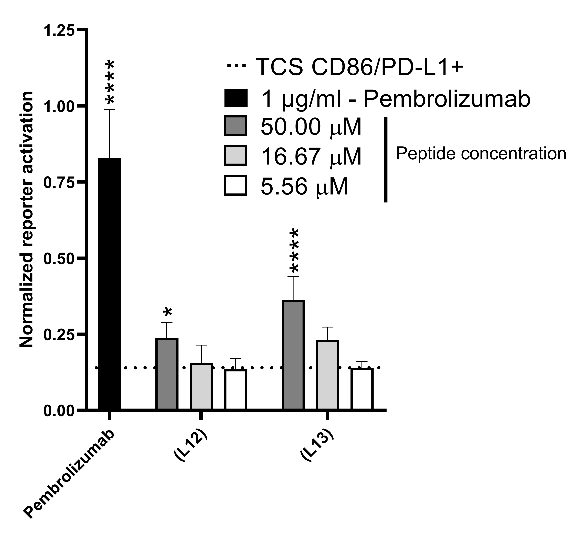** | 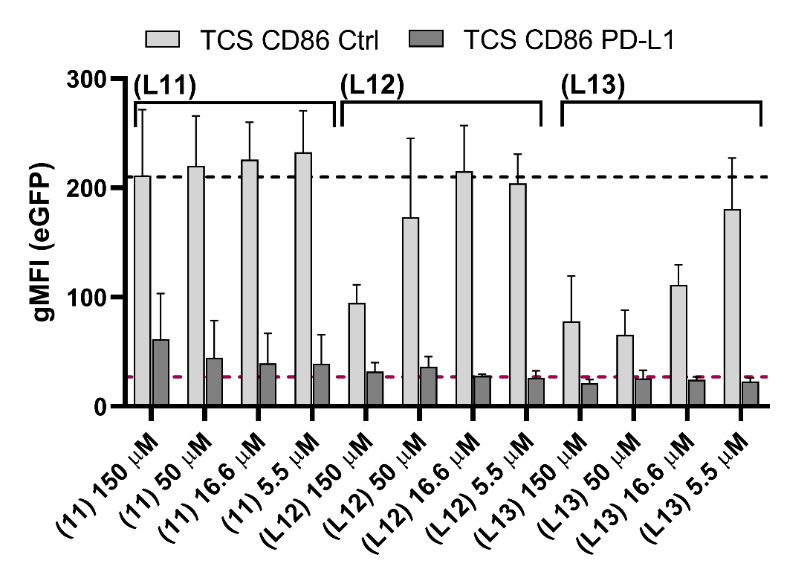 |

**Figure S4**. A) The inhibitory properties of the peptides in the functional cellular assay. The PD-1 reporter cells were stimulated with TCS CD86 expressing PD-L1 in the absence or presence of the peptides. The inhibitory properties of the peptides were measured by the eGFP expression using flow cytometry. A) The results were normalised to gMFI eGFP values of PD-1 reporter cells stimulated with TCS CD86 (stimulation without PD-1 inhibition) treated by the peptides. The dotted line shows the normalised eGFP expression level from the coculture of the PD-1 reporter cells with TCS CD86/PD-L1 without peptides or antibodies. B) The non-normalized data are shown raw. The grey dotted back lines correspond to the PD-1 reporter cell stimulated with TCS CD86 in the absence of the peptides. The purple dotted back line corresponds to the PD-1 reporter cell stimulated with TCS CD86/PD-L1 in the absence of the peptides. Results are shown for three experiments performed independently in duplicate. Data are depicted as mean with SD. Statistical analysis was performed using one-way ANOVA followed by the Dunnet’s post-hoc test. ****: p < 0.0001, ***: p < 0.001, **: p < 0.01, *: p < 0.05.

***Table S4.*** *Proton chemical shifts for peptide (****L11****).*

| **Residue** | **Proton chemical shifts /ppm** | | | |
| --- | --- | --- | --- | --- |
|  | **HN** | **Hα** | **Hβ** | **Others** |
| Val1 | 8.14 | 4.09 | 2.05 | γ-CH_3_ 0.94, CH_3_CO 2.04 |
| Cys2 | 8.57 | 4.79 | 3.06, 3.10 |  |
| Arg3 | 8.47 | 4.37 | 1.76, 1.85 | Hγ 1.61, Hδ 3.16, ε-NH 7.18 |
| Abu4 | 8.15 | 4.25 | 1.70, 1.79 | γ-CH_3_ 0.89 |
| Nle5 | 8.15 | 4.33 | 1.72 | Hγ 1.59, Hδ 1.24, ε-CH_3_ 0.82 |
| Ile6 | 8.10 | 4.18 | 1.80 | Hγ1 1.12, 1.41, δ1-CH_3_ 0.81, γ2-CH_3_ 0.82 |
| Ser7 | 8.21 | 4.47 | 3.78 |  |
| Tyr8 | 8.22 | 4.48 | 2.97, 3.04 | Hδ 7.08, Hε 6.81 |
| Gly9 | 8.25 | 3.83, 3.91 |  |  |
| Gly10 | 8.06 | 3.91, 3.98 |  |  |
| Ala11 | 8.18 | 4.30 |  | β-CH_3_ 1.32 |
| Asp12 | 8.38 | 4.71 | 2.73, 2.81 |  |
| Tyr13 | 8.04 | 4.52 | 2.95, 3.02 | Hδ 7.05, Hε 6.78 |
| Lys14 | 8.19 | 4.30 | 1.73 | Hγ 1.32, Hδ 1.63, Hε 2.96, ζ-NH_2_ 7.53 |
| Arg15 | 8.21 | 4.37 | 1.75, 1.85 | Hγ 1.64, Hδ 3.19, ε-NH 7.18 |
| Cys16 | 8.45 | 4.82 | 3.02, 3.22 |  |
| Thr18 | 8.19 | 4.33 | 4.23 | γ2-CH_3_ 1.17, CO-NH_2_ 7.19, 7.57 |


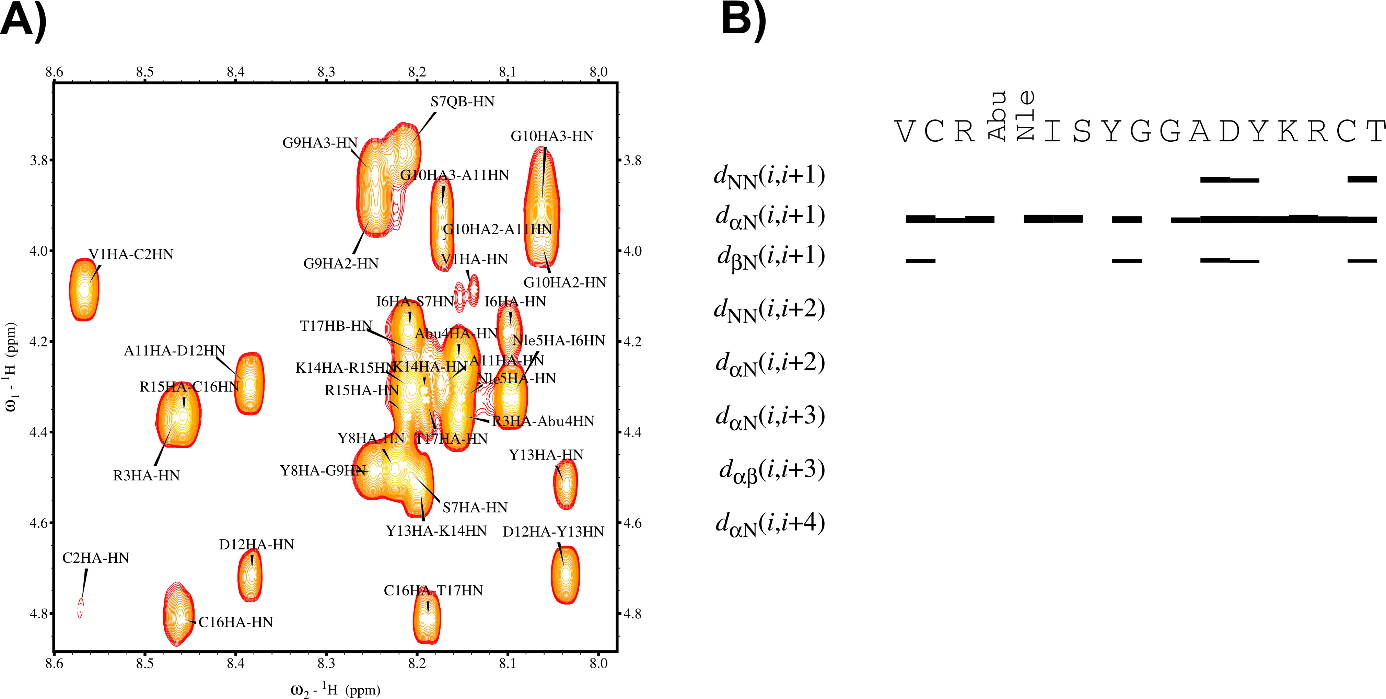


***Figure S5.*** *(****A****) HN-Hα region of NOESY spectrum of peptide (****L11****) recorded with a mixing time 200 ms.(****B****) Sequence plots of NOESY distance constraints of studied peptide (****L11****).*

**
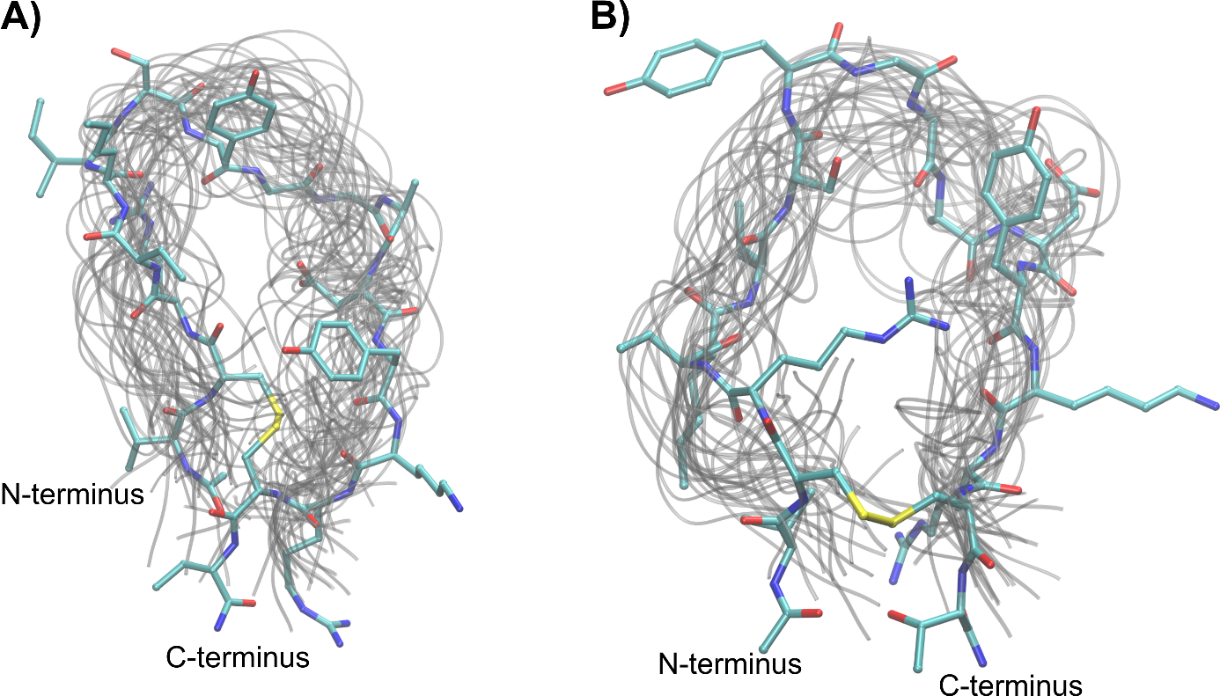
**

***Figure S6.*** *Superimposed conformations of the representative conformational families of* ***(L11)****. (****A****) Family 1 with 42 structures, the root-mean square deviation for the backbone atoms from residue 2 to 16 for this family of the structures is RMSD_2-16_=3.25±0.38 Å relative to the lowest-energy conformation and RMSD_2-16_=2.07±0.28 Å relative to the average conformation (****B****) Family 2 with 31 structures, the root-mean square deviation for the backbone atoms from residue 2 to 16 for this family of the structures is RMSD_2-16_=2.81±0.50 Å relative to the lowest-energy conformation, and RMSD_2-16_=2.01±0.32 Å relative to the average conformation.*


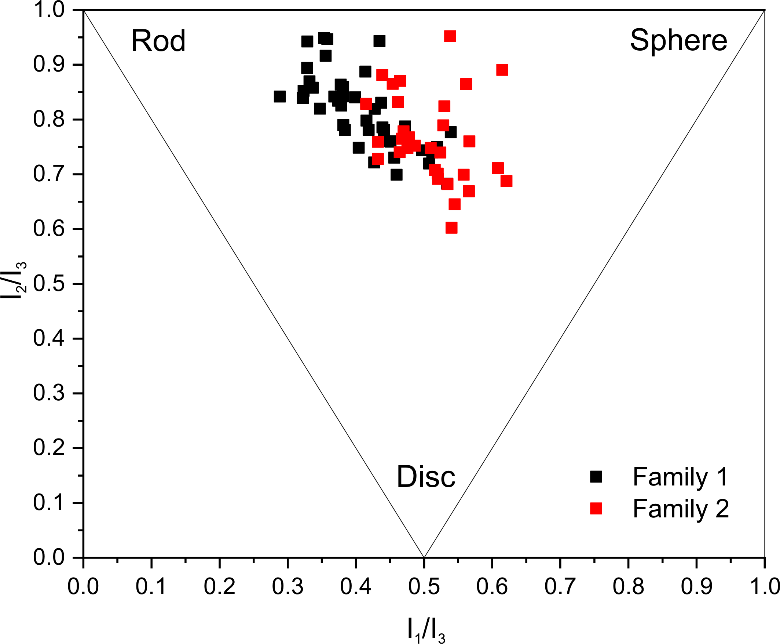


***Figure S7.*** *Principal moments of inertia (PMI) analysis of the* ***(L11)*** *structures from two representative conformational families.*

*
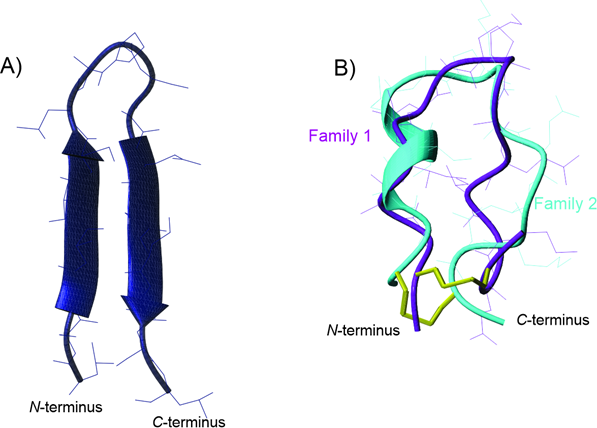
*

***Figure S8.*** *The structure of the PD-L1(111-127) fragment A) trimmed from the PD-1/PD-L1 complex (PDB code: 4ZQK); B) determined by NMR for two of the most important conformation families of the peptide (****L11****). Disulfide bonds are marked in yellow.*

**
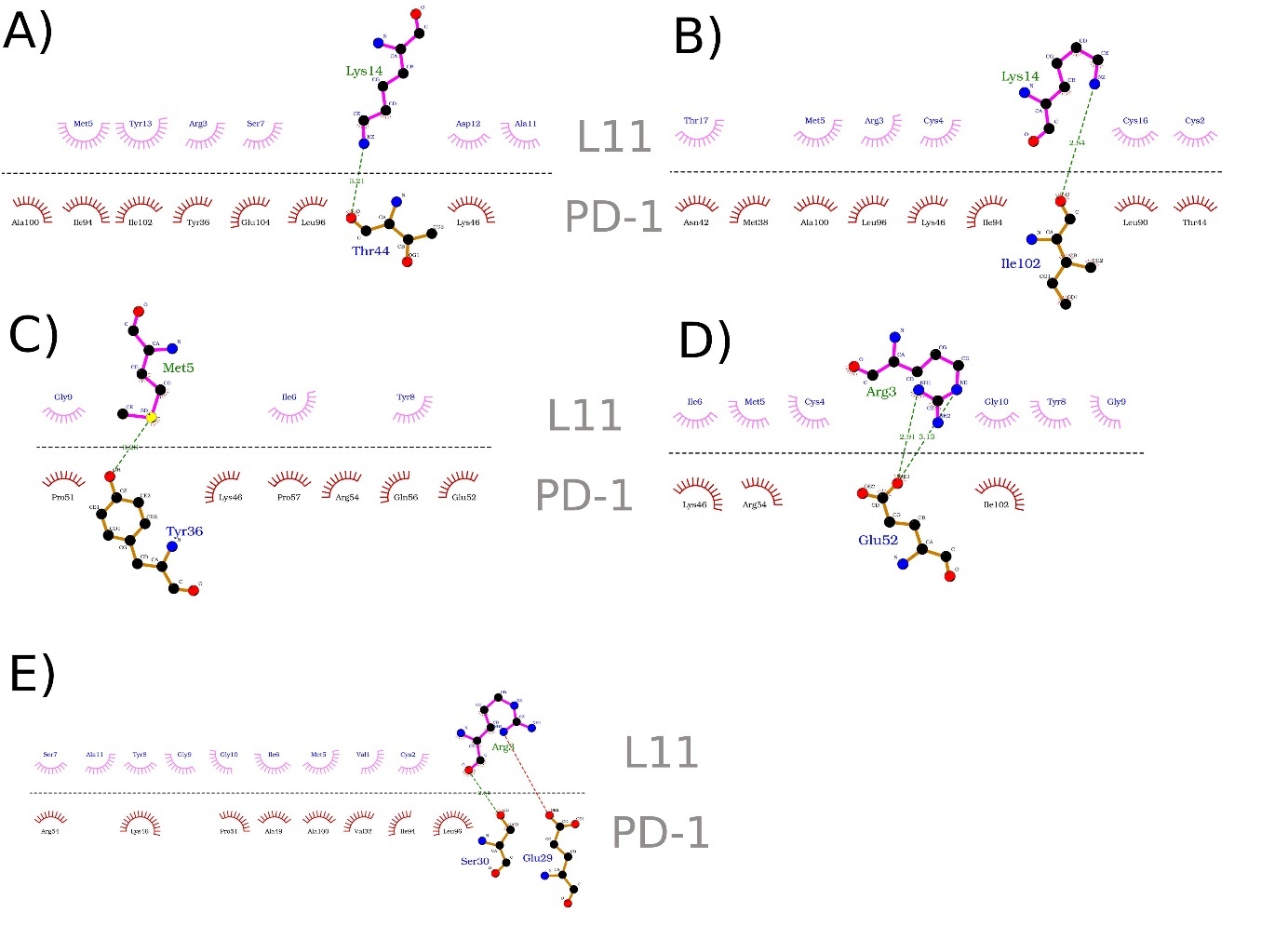
Figure S9.** The contact map of the selected binding modes: (A) trimmed from PD-1/PD-L1 complex (PDB code: 4ZQK). Structures B-E show the complexes of peptide (**L11**) obtained from docking NMR structures to PD-1 using the UNRES force field, (B) cluster from family 1 (lowest energy), (C) cluster from family 1 (centroid), (D) cluster from family 2 (lowest energy), (E) cluster from family 2 (centroid). Compare with Figure 8 of the main manuscript.

**Table S5.** Peptides and peptidomimetics potentially interacting with the PD-1 protein as described in the literature. D - amino acids are marked with a lowercase letter. NB - no binding; ND - no data; JD - no name given by the authors.

| No. | Peptide name | Amino acid sequence | Affinity  (K_D_) [µM] | Method of peptide designing | The year of publication |
| --- | --- | --- | --- | --- | --- |
| 1 | Ar5Y_1[3] | FNWDYSWKSERLKEAYDL | 3.39 | Computational methods | 2016 |
| 2 | Ar5Y_2[3] | FNWDYSLEELREKAKYK | 3.14 |  |  |
| 3 | Ar5Y_3[3] | TEKDYRHGNIRMKLAYDL | 3.13 |  |  |
| 4 | Ar5Y_4[3] | GNWDYNSQRAQLYNQ | 1.38 |  |  |
| 5 | Ar5Y_4 W3A[3] | GNADYNSQRAQLYNQ | 8.08 |  |  |
| 6 | Ar5Y_4 D4A[3] | GNWAYNSQRAQLYNQ | 18.94 |  |  |
| 7 | Ar5Y_4 Y5A[3] | GNWDANSQRAQLYNQ | 20.15 |  |  |
| 8 | Ar5Y_4 R9A[3] | GNWDYNSQAAQLYNQ | 21.20 |  |  |
| 9 | Ar5Y_4 Y13A[3] | GNWDYNSQRAQLANQ | 10.23 |  |  |
| 10 | Ar3_ref[3] | ADYK | 370.4 |  |  |
| 11 | Ar3_1[3] | WDYD | 22.3 |  |  |
| 12 | Ar4_1[3] | GIDYEERWK | 28.28 |  |  |
| 13 | Ar4_2[3] | LDYDGRLSQ | 83.90 |  |  |
| 14 | Ar5M_1[3] | LDYGDKREGQMAE | 21.60 |  |  |
| 15 | Ar5M_2[3] | LDYVNRRKMYQ | 3.32 |  |  |
| 16 | PDLong1  (PD-L1 9-28)[4] | FMTYWHLLNAFTVTVPKDL | - | Rational design based on PD-L1 protein amino acid sequence | 2016 |
| 17 | PDLong2  (PD-L1 242-264)[4] | VILGAILLCLGVALTFIFRLRKG | - |  |  |
| 18 | PL120_L_-131_L_[5] | GADYKRITVKVN | ND | Rational design based on PD-L1 protein amino acid sequence | 2018 |
| 19 | PD64-78[5] | VLNWYRMSPSNQTDK | ND | Rational design based on PD-1 protein amino acid sequence |  |
| 20 | WANG-003[6] | KRWWR | 3.3 | Computational methods | 2019 |
| 21 | WANG-004[6] | FRWWR | 1.6 |  |  |
| 22 | WANG-005[6] | RRWQWR | 5.1 |  |  |
| 23 | WANG-006[6] | YVAM | NB |  |  |
| 24 | WANG-007[6] | YVAE | NB |  |  |
| 25 | YT-16  PDL1(112-127) C114-C125[7] | YRCMISYGGADYKCIT (C-C) | 0.0178 | Rational design based on PD-L1 protein amino acid sequence/ Computational methods | 2019 |
| 26 | PDL1(112-127) C114-C125; Y118-P[7] | YRCMISPGGADYKCIT | - |  |  |
| 27 | PDL1(112-127) C114-C125; D122-E[7] | YRCMISYGGAEYKCIT | - |  |  |
| 28 | PDL1(112-127) C114-C125; Y118-P, D122-E[7] | YRCMISPGGAEYKCIT | - |  |  |
| 29 | PDL1(112-127) C114-C125; S117-T[7] | YRCMITYGGGDYKCIT | - |  |  |
| 30 | PDL1(112-127) C114-C125; S117-T; Y118-P[7] | YRCMITPGGGDYKCIT | - |  |  |
| 31 | PDL1(112-127) C114-C125;S117T;D122-E[7] | YRCMITYGGGEYKCIT | - |  |  |
| 32 | PDL1(112-127) C114-C125; S117-T; Y118-P; D122-E[7] | YRCMITPGGGEYKCIT |  |  |  |
| 33 | DS-I[8]  54-68 | IVYWEMEDKNIIQFV | - | Rational design based on PD-L1 protein amino acid sequence/ Computational methods | 2019 |
| 34 | DS-II[8]  110-132 | GVYRCMISYGGADYKRITVKVNA | 109 |  |  |
| 35 | DS-II  [C111–C127][8]  110-132 | GCYRCMISYGGADYKRICVKVNA | 28 |  |  |
| 36 | ΔDS-II  [C111–C127][8]  111-127 | CYRCMISYGGADYKRIC | 17.5 |  |  |
| 37 | ΔDS-II  [C110–C128][8]  110-128 | CVYRCMISYGGADYKRITC | 11.6 |  |  |
| 38 | Inhibitor 1[9]  PD-L1(112-128) | YRCMISYGGADYKRITV | - | Protein painting | 2019 |
| 39 | Inhibitor 2[9]  Similar to Inhibitor 1, cyclized via Cys–Cys bond | CYRAMISYGGADYKRITC | - |  |  |
| 40 | Inhibitor 3[9]  PD-L1: Leu-15, Lys-124 to Ala-121, Pro-24, Ala-18 to Thr-22 | LKYDAPAFTVT | - |  |  |
| 41 | Inhibitor 4 [9]  Similar to Inhibitor 3 cyclized via Cys–Cys bond | CLKYDAPAFTVTC | - |  |  |
| 42 | CLP001[10] | HYPFRPHANQAS | 0.534 | Phage display | 2019 |
| 43 | CLP002[10] | WHRSYYTWNLNT | 0.366 |  |  |
| 44 | CLP003[10] | WHFSYNWRWLPP | 0.117 |  |  |
| 45 | CLP004[10] | DYHDPSLPTLRK | 0.544 |  |  |
| 46 | P1.1[11]  PD-L1(113-126) | RCMICYGGADYKRI | 3.66 | Rational design based on PD-L1 protein amino acid sequence | 2020 |
| 47 | P1.2[11] | RCMISYpGADYKRI | 83.05 |  |  |
| 48 | P1.3[11] | RTMIWYpGAWYKRI | 1.80 |  |  |
| 49 | P1.4[11] | RTCITYpGADYCRI | 474 |  |  |
| 50 | P2.1[11] | RTMIWYpPAWYKRI | 29.20 |  |  |
| 51 | P2.2[11] | RTMIWYAAAWYKRI | 55.20 |  |  |
| 52 | P2.3[11] | RTMIWYGpAWYKRI | 129.00 |  |  |
| 53 | Q1  PD-L1(110-129) | GVYRCMISYGGADYKRITVKV | - | Rational design based on PD-L1 protein amino acid sequence/ Computational methods | 2020 |
| 54 | Pep1[12] | LETWFGKEILVKT | 140.0 |  |  |
| 55 | Pep2[12] | VFELRHSKRKDSRTVY | 127.0 |  |  |
| 56 | Pep3[12] | TSEVDNGVGKPQKHS | 81.1 |  |  |
| 57 | Pep4[12] | TFKVEDRYGQGQQILD | 22.7 |  |  |
| 58 | Pep6[12] | KSKAVNRVSQESEM | 66.4 |  |  |
| 59 | Pep9[12] | LEYWSSGSTTMYGL | 36.4 |  |  |
| 60 | Pep10[12] | LERHDFGDGRARYEE | 39.0 |  |  |
| 61 | Pep14[12] | QYRCNGTSSKGSDQAIITLRV | 10.9 |  |  |
| 62 | PD-i1[13] | [TnTDYnPtLl] | - | Computational methods | 2020 |
| 63 | PD-i2[13] | [vpTSYSpDDv] | - |  |  |
| 64 | PD-i3[13] | [TMYLerRYpD] | 102 |  |  |
| 65 | PD-i4[13] | [LddRYpnLPM] | - |  |  |
| 66 | PD-i5[13] | [DDqSWNiPfs] | - |  |  |
| 67 | PD-i6[13] | [WwVpEAkD] | 30 |  |  |
| 68 | PD-i7[13] | [NsDYTyPF] | - |  |  |
| 69 | IMB-P6-10[14] | LTCSLAPNIISAL | - | hPRDX5‑based peptide | 2020 |
| 70 | WQ-20[15] | - | 305 | Phage display | 2020 |
| 71 | QP-20[15] | - | 10.7 |  |  |
| 72 | HD-20[15] | - | 3.41 |  |  |
| 73 | SQ-20[15] | - | 5.16 |  |  |
| 74 | C8[16] | [CKWYRPSEC] | 0.64 | Phage display | 2020 |
| 75 | P-S2[17] | SSHFAYLGAYSS | - | Phage display | 2020 |
| 76 | P-T3[17] | EWSSWGYRDQQT | - |  |  |
| 78 | P-V1[17] | FSPRPGLHSYGV | - |  |  |
| 79 | P-F4[17] | FSGTVTTAGLLF | 0.119 |  |  |
| 80 | P-H5[17] | STTSNFFGALVH | - |  |  |
| 81 | nABP284[18] | SRLKEIANSPTQFWRMVARNTLGNGAKQSLNIEHARL | 11.8 | Phage display/ computational methods | 2021 |
| 82 | nABPD1[18] | SHHHRLSRLKEIANSPTQFWRMVARNTLGNGAKQSLNIEHARL | 0.0119 |  |  |
| 83 | JD1[19] | RRWWRR | 4.01 | Peptide library | 2021 |
| 84 | JD2[19] | RRQWFW | 4.38 |  |  |
| 85 | Peptide-1[20]  PD-L1(112-127)(^C114-R125C)^ | YRCMISYGGADYKCIT | - |  | 2022 |
| 86 | MN1.1[21]  PD-L1(112-128) | YRCMISYGGADYKRITV | - | Computational methods | 2023 |
| 87 | MN1.2[21]  PD-L1(112-128)(^Y112C-V128C)^ | CRCMISYGGADYKRITC | - |  |  |
| 88 | MN1.3[21] | - | - |  |  |
| 89 | MN1.4[21] | CRAMISYGGADYK-(N-Me-Arg)-IC | 2 |  |  |
| 90 | MN1.5[21] | CRANleISYGGADYK-(N-Me-Arg)-IC | - |  |  |
| 91 | PBP[22] | SGQQRTSADCWEDHGWGSGGSK | 17.44 | Bacterial surface display technology | 2023 |

***Table S6****. Peptides and peptidomimetics potentially interacting with the PD-L1 protein as described in the literature. D - amino acids are marked with a lowercase letter. NB - no binding; ND - no data; JD - no name given by the authors.*

| No. | Peptide name | Amino acid sequence | Affinity  (K_D_) [µM] | Method of peptide designing | The year of publication |
| --- | --- | --- | --- | --- | --- |
| 1 | AUNP-12[23–25] (compound 8) | SNTSESF-NH_2_  SNTSESFKFRVTQLAPKAQIKE-NH_2_ | - | Rational design based on PD-1 protein amino acid sequence | 2014 |
| 2 | P1[26]  PD-1(73-78) | SNQTDK | - | Rational design based on PD-1 protein amino acid sequence | 2012 |
| 3 | P2[26] - control | ADTKRI | - |  |  |
| 4 | L8[27] | SLPSTTTMRLTS | - | ND | 2014 |
| 5 | JD[28] | ANGSRLV | - | ND | 2014 |
| 6 | S10[29] | WSHGGHQHFIRF | - | ND | 2014 |
| 7 | p101[30–32] | FFIVIRDRVFRGScc | - | ND | 2014 |
| 8 | PPA-1[33] | nkskptdrqyhf | 0.51 | Phage display/mirror-image phage display | 2015 |
| 9 | PPA-2[33] | khahhthnlrlp | 1.13 |  |  |
| 10 | PPA-3[33] | aakmgdhlhggq | NB |  |  |
| 11 | PPA-4[33] | mrnrerypkpyy | 22 |  |  |
| 12 | PPA-5[33] | tlyqrpstnler | NB |  |  |
| 13 | PPA-1 - scramble[33] | rhtndysqfypk | NB |  |  |
| 14 | TPP-1[34] | SGQYASYHCWCWRDPGRSGGSK | 0.095 | Random bacterial surface display method | 2018 |
| 15 | IMB-P6-10[14] | LTCSLAPNIISAL | - | In silico proteolysis of hPRDX5/rational design | 2019 |
| 16 | IO103[35]  PD-L1(9-27) | FMTYWHLLNAFTVTVPKDL | - | Rational design based on PD-L1 protein amino acid sequence | 2019 |
| 17 | IO101[35]  PD-L1(15-23) | LLNAFTVTV | - |  |  |
| 18 | Inhibitor 5[9]  PD-1(65-81) | LNWYRMSPSNQTDKLAA | - | Protein painting | 2019 |
| 19 | Inhibitor 6[9]  Similar to Inhibitor 5, cyclized via Cys–Cys bond | CLNWYRMSPSNQTDKLAC | - |  |  |
| 20 | Inhibitor 7[9]  PD-1(125-135) | AISLAPKAQIK | - |  |  |
| 21 | Inhibitor 7[9]  Similar to Inhibitor 7, cyclized via Cys–Cys bond | CAISLAPKAQIKC | - |  |  |
| 22 | PD-L1Pep-1[36] | CLQKTPKQC | 0.373 | Phage display | 2020 |
| 23 | PD-L1Pep-2[36] | CVRARTR | 0.281 |  |  |
| 24 | HS1[37]  PD-1(121-137) | YCGAISLAPKAQIKES | - | Rational design based on PD-1 protein amino acid sequence | 2021 |
| 25 | HS2[37] | CGAISLAPKAQIKES | - |  |  |
| 26 | HS3[37] | CGAISLAPKLQIKES | - |  |  |
| 27 | HS4[37] | GAISLAPKLQINE | - |  |  |
| 28 | HS5[37] | GAISLAPKLQINe | - |  |  |
| 29 | HS6[37] | H_2_N-GAISLAPKLQINEG-CO (C,N-terminal lactam peptide) | - |  |  |
| 30 | HS7[37] | GAISLAPKLQIND | - |  |  |
| 31 | OPBP-1[38] | GQSEHHMRVYSF | 0.667 | Liquid-phase phage display | 2021 |
| 32 | CLP-2[39] | WHRSYYTWNLNT | - | Phage display/SAR studies | 2022 |
| 33 | C7[39] | CHRSYYTWNLNC | - |  |  |
| 34 | C12[39] | CHRSYYTWNCNT | - |  |  |
| 35 | Peptide-2[20] | CGIQDTNSKKQSDTHLEET | - | AI-directed peptide design | 2022 |
| 36 | PD-L1ip3[40] | GTRLKPLIICVQWPGL | 33 | Computational design | 2022 |
| 37 | P[41] | CPLGVRGKGGGNYSKPTDRQYHF | - | - | 2022 |
| 38 | PDP[42] | CHEHEKFEQR-(PEG3)-nyskptdrqyhfrr | - | - | 2023 |
| 39 | A11[43] | EYVQTVKSSKG | - | Based on Annexin 1 | 2023 |
| 40 | PD-L1(23-31)[44] | VPK DLY VVE | - | Rational design | 2023 |
| 41 | PP[45] | nyskptdrqyhf | - | - | 2023 |
| 42 | CSBP[46] | ldvflyse | 7.53 | Phage display | 2023 |
| 43 | 4F[47] | DWFKAFYDKVAEKFKEAF | - | Rational design based on apolipo-  protein A-I (apoA-I)/computational design | 2023 |
| 44 | 4F-8[47] | GDWFKAYYDKVAEG | 412.5 |  |  |
| 45 | 4F-16[47] | SHFSASYDKYAEKF | 49.4 |  |  |
| 46 | 4F-30[47] | DWIKANYDETAEYNKEAK | 419 |  |  |
| 47 | 4F-32[47] | DWFKAFYDKINETYNK | 0.017 |  |  |

[1] M. Bojko, K. Węgrzyn, E. Sikorska, M. Kocikowski, M. Parys, C. Battin, P. Steinberger, M.M. Kogut, M. Winnicki, A.K. Sieradzan, M. Spodzieja, S. Rodziewicz-Motowidło, Design, synthesis and biological evaluation of PD-1 derived peptides as inhibitors of PD-1/PD-L1 complex formation for cancer therapy, Bioorg. Chem. 128 (2022). https://doi.org/10.1016/j.bioorg.2022.106047.

[2] K.M. Zak, R. Kitel, S. Przetocka, P. Golik, K. Guzik, B. Musielak, A. Dömling, G. Dubin, T.A. Holak, Structure of the Complex of Human Programmed Death 1, PD-1, and Its Ligand PD-L1, Structure. 23 (2015) 2341–2348. https://doi.org/10.1016/j.str.2015.09.010.

[3] L. Zhu, X. Wang, H. Li, Z. Zhao, X. Liu, J. Lyu, J. Meng, Q. Li, Z. He, L. Quan, Discovery of peptide inhibitors targeting human programmed death 1 (PD-1) receptor, Oncotarget. 7 (2016). https://doi.org/10.18632/oncotarget.11274.

[4] S. Munir Ahmad, E. Martinenaite, M. Hansen, N. Junker, T.H. Borch, Ö. Met, M. Donia, I.M. Svane, M.H. Andersen, PD-L1 peptide co-stimulation increases immunogenicity of a dendritic cell-based cancer vaccine, Oncoimmunology. 5 (2016). https://doi.org/10.1080/2162402X.2016.1202391.

[5] R.J. Boohaker, V. Sambandam, I. Segura, J. Miller, M. Suto, B. Xu, Rational design and development of a peptide inhibitor for the PD-1/PD-L1 interaction, Cancer Lett. 434 (2018) 11–21. https://doi.org/10.1016/j.canlet.2018.04.031.

[6] Y. Wang, H. Guo, Z. Feng, S. Wang, Y. Wang, Q. He, G. Li, W. Lin, X.Q. Xie, Z. Lin, PD-1-Targeted Discovery of Peptide Inhibitors by Virtual Screening, Molecular Dynamics Simulation, and Surface Plasmon Resonance, Molecules. 24 (2019) 1–15. https://doi.org/10.3390/molecules24203784.

[7] A.B. Abbas, B. Lin, C. Liu, A. Morshed, J. Hu, H. Xu, Design and synthesis of A PD-1 binding peptide and evaluation of its anti-tumor activity, Int. J. Mol. Sci. 20 (2019) 1–19. https://doi.org/10.3390/ijms20030572.

[8] K. Zhou, J. Lu, X. Yin, H. Xu, L. Li, B. Ma, Structure-based derivation and intramolecular cyclization of peptide inhibitors from PD-1/PD-L1 complex interface as immune checkpoint blockade for breast cancer immunotherapy, Biophys. Chem. 253 (2019). https://doi.org/10.1016/j.bpc.2019.106213.

[9] A. Haymond, D. Dey, R. Carter, A. Dailing, V. Nara, P. Nara, S. Venkatayogi, M. Paige, L. Liotta, A. Luchini, Protein painting, an optimized MS-based technique, reveals functionally relevant interfaces of the PD-1/PD-L1 complex and the YAP2/ZO-1 complex, J. Biol. Chem. 294 (2019) 11180–11198. https://doi.org/10.1074/jbc.RA118.007310.

[10] H. Liu, Z. Zhao, L. Zhang, Y. Li, A. Jain, A. Barve, W. Jin, Y. Liu, J. Fetse, K. Cheng, Discovery of low-molecular weight anti-PD-L1 peptides for cancer immunotherapy, J. Immunother. Cancer. 7 (2019) 1–14. https://doi.org/10.1186/s40425-019-0705-y.

[11] K. Wang, Y. Song, Y. Su, Y. Liang, L. Wang, Effect of the hairpin structure of peptide inhibitors on the blockade of PD-1/PD-L1 axis, Biochem. Biophys. Res. Commun. 527 (2020) 453–457. https://doi.org/10.1016/j.bbrc.2020.04.018.

[12] P. Zhang, C. Li, X. Ji, M. Gao, S. Lyu, X. Dai, J. Du, In silico screening and surface plasma resonance-based verification of programmed death 1-targeted peptides, Chem. Biol. Drug Des. 95 (2020) 332–342. https://doi.org/10.1111/cbdd.13647.

[13] S. Guardiola, M. Varese, X. Roig, J. Garcia, E. Giralt, A Target-Based Method for Designing Heterochiral Cyclic Peptide Binders: De Novo Inhibitors of the PD-1/PD-L1 Interaction, (n.d.). https://doi.org/10.26434/chemrxiv.11663337.v1.

[14] S. Zou, J. Liu, Z. Sun, X. Feng, Z. Wang, Y. Jin, Z. Yang, Discovery of hPRDX5-based peptide inhibitors blocking PD-1/PD-L1 interaction through in silico proteolysis and rational design, Cancer Chemother. Pharmacol. 85 (2020) 185–193. https://doi.org/10.1007/s00280-019-03995-z.

[15] V. Kotraiah, T.W. Phares, C.D. Browne, J. Pannucci, M. Mansour, A.R. Noe, K.D. Tucker, J.M. Christen, C. Reed, A. MacKay, G.M. Weir, R. Rajagopalan, M.M. Stanford, C.S. Chung, A. Ayala, J. Huang, M. Tsuji, G.M. Gutierrez, Novel Peptide-Based PD1 Immunomodulators Demonstrate Efficacy in Infectious Disease Vaccines and Therapeutics, Front. Immunol. 11 (2020) 1–17. https://doi.org/10.3389/fimmu.2020.00264.

[16] W. Zhai, X. Zhou, M. Zhai, W. Li, Y. Ran, Y. Sun, J. Du, W. Zhao, L. Xing, Y. Qi, Y. Gao, Blocking of the PD-1/PD-L1 interaction by a novel cyclic peptide inhibitor for cancer immunotherapy, Sci. China Life Sci. 64 (2021) 548–562. https://doi.org/10.1007/s11427-020-1740-8.

[17] H. Tao, L. Cheng, L. Liu, H. Wang, Z. Jiang, X. Qiang, L. Xing, Y. Xu, X. Cai, J. Yao, M. Wang, Z. Qiu, A PD-1 peptide antagonist exhibits potent anti-tumor and immune regulatory activity, Cancer Lett. 493 (2020) 91–101. https://doi.org/10.1016/j.canlet.2020.08.009.

[18] Y. Chen, H. Huang, Y. Liu, Z. Wang, Q. Wang, Y. Zhang, H. Wang, Engineering a High-Affinity PD-1 Peptide for Optimized Immune Cell-Mediated Tumor Therapy, Cancer Res. Treat. (2021) 1–31. https://doi.org/10.4143/crt.2021.424.

[19] G. Li, H. Guo, L. Zhao, H. Feng, H. He, Y. Chen, Y. Wang, Z. Lin, Discovery of modulators for the PD-1/PD-L1 interaction by molecular simulation and bioassay, New J. Chem. 45 (2021) 18497–18508. https://doi.org/10.1039/D1NJ02030G.

[20] M.-Y. Dai, Y.-Y. Shi, A.-J. Wang, X.-L. Liu, M. Liu, H.-B. Cai, High-potency PD-1/PD-L1 degradation induced by Peptide-PROTAC in human cancer cells, Cell Death Dis. 13 (2022) 924. https://doi.org/10.1038/s41419-022-05375-7.

[21] R. Carter, F. Alanazi, A. Sharp, J. Roman, A. Luchini, L. Liotta, M. Paige, A.M. Brown, A. Haymond, Identification of the functional PD-L1 interface region responsible for PD-1 binding and initiation of PD-1 signaling, J. Biol. Chem. 299 (2023) 105353. https://doi.org/10.1016/j.jbc.2023.105353.

[22] Y. Qin, X. Meng, L. Li, C. Liu, F. Gao, X. Yuan, Y. Huang, Y. Zhu, Develop a PD-1-blockade peptide to reinvigorate T-cell activity and inhibit tumor progress, Eur. J. Pharmacol. 960 (2023) 176144. https://doi.org/10.1016/j.ejphar.2023.176144.

[23] P.G.N. Sasikumar, M. Ramachandra, S.K. Vadlamani, K.R. Vemula, L.K. Satyam, K. Subbarao, R.K. Shrimali, S. Kandepu, IMMUNOSUPPRESSION MODULATING COMPOUNDS, 2011.

[24] P.G. Sasikumar, L.K. Satyam, R.K. Shrimali, K. Subbarao, R. Ramachandra, S. Vadlamani, A. Reddy, A. Kumar, A. Srinivas, S. Reddy, S. Gopinath, D.S. Samiulla, M. Ramachandra, Abstract 2850: Demonstration of anti-tumor efficacy in multiple preclinical cancer models using a novel peptide inhibitor (Aurigene-012) of the PD1 signaling pathway, in: Cancer Res., American Association for Cancer Research (AACR), 2012: pp. 2850–2850. https://doi.org/10.1158/1538-7445.am2012-2850.

[25] P.G. Sasikumar, R.K. Ramachandra, S. Adurthi, A.A. Dhudashiya, S. Vadlamani, K. Vemula, S. Vunnum, L.K. Satyam, D.S. Samiulla, K. Subbarao, R. Nair, R. Shrimali, N. Gowda, M. Ramachandra, A rationally designed peptide antagonist of the PD-1 signaling pathway as an immunomodulatory agent for cancer therapy, Mol. Cancer Ther. 18 (2019) 1081–1091. https://doi.org/10.1158/1535-7163.MCT-18-0737.

[26] F. Wang, J. Ma, J. Liu, H. Jin, D. Huang, Synthetic small peptides acting on B7H1 enhance apoptosis in pancreatic cancer cells, Mol. Med. Rep. 6 (2012) 553–557. https://doi.org/10.3892/mmr.2012.970.

[27] A kind of PD-1 albumen extracellular fragment affinity peptide L8 and application thereof, 2014.

[28] PD-L1 IgV affinity peptide with antineoplastic activity, and preparation method and application of thereof, 2014.

[29] PD-L1 IgV affinity peptide S10 with antitumor activity, 2014.

[30] P.M.S. M. Miller, C. Mapelli, M. P. Allen, M. S. Bowsher, K. M. Boy, E. P. Gillis, D. R. Langley, E. Mull, M. A. Poirier, N. Sanghvi, L.-Q. Sun, D. J. Tenney, K.-S. Yeung, J. Zhu, P. C. Reid, WO2014151634A1 - Macrocyclic inhibitors of the pd-1/pd-l1 and cd80(b7-1)/pd-l1 protein/protein interactions - Google Patents, 2014.

[31] K. Magiera‐Mularz, K. Kuska, L. Skalniak, P. Grudnik, B. Musielak, J. Plewka, J. Kocik, M. Stec, K. Weglarczyk, D. Sala, B. Wladyka, M. Siedlar, T.A. Holak, G. Dubin, Macrocyclic Peptide Inhibitor of PD‐1/PD‐L1 Immune Checkpoint, Adv. Ther. 4 (2021) 2000195. https://doi.org/10.1002/adtp.202000195.

[32] E. Zyla, B. Musielak, T.A. Holak, G. Dubin, Structural Characterization of a Macrocyclic Peptide Modulator of the PD-1/PD-L1 Immune Checkpoint Axis, Molecules. 26 (2021) 4848. https://doi.org/10.3390/molecules26164848.

[33] H.-N. Chang, B. Liu, Y. Qi, Y. Zhou, Y.-P. Chen, K.-M. Pan, W.-W. Li, X.-M. Zhou, W.-W. Ma, C.-Y. Fu, Y.-M. Qi, L. Liu, Y.-F. Gao, Blocking of the PD‐1/PD‐L1 Interaction by a <scp>D</scp> ‐Peptide Antagonist for Cancer Immunotherapy, Angew. Chemie Int. Ed. 54 (2015) 11760–11764. https://doi.org/10.1002/anie.201506225.

[34] C. Li, N. Zhang, J. Zhou, C. Ding, Y. Jin, X. Cui, K. Pu, Y. Zhu, Peptide Blocking of PD-1/PD-L1 interaction for cancer immunotherapy, Cancer Immunol. Res. 6 (2018) 178–188. https://doi.org/10.1158/2326-6066.CIR-17-0035.

[35] S. Munir, M.T. Lundsager, M.A. Jørgensen, M. Hansen, T.H. Petersen, C.M. Bonefeld, C. Friese, Ö. Met, P. thor Straten, M.H. Andersen, Inflammation induced PD-L1-specific T cells, Cell Stress. 3 (2019) 319–327. https://doi.org/10.15698/cst2019.10.201.

[36] S. Gurung, F. Khan, G.R. Gunassekaran, J. Do Yoo, S.M. Poongkavithai Vadevoo, U. Permpoon, S.-H. Kim, H.-J. Kim, I.-S. Kim, H. Han, J. Park, S. Kim, B. Lee, Phage display-identified PD-L1-binding peptides reinvigorate T-cell activity and inhibit tumor progression, Biomaterials. (2020) 119984. https://doi.org/10.1016/j.biomaterials.2020.119984.

[37] A. Orafaie, H. Sadeghian, A.R. Bahrami, H. Rafatpanah, M.M. Matin, Design, synthesis and evaluation of PD-L1 peptide antagonists as new anticancer agents for immunotherapy, Bioorg. Med. Chem. 30 (2021) 115951. https://doi.org/10.1016/j.bmc.2020.115951.

[38] W. Li, X. Zhu, X. Zhou, X. Wang, W. Zhai, B. Li, J. Du, G. Li, X. Sui, Y. Wu, M. Zhai, Y. Qi, G. Chen, Y. Gao, An orally available PD-1/PD-L1 blocking peptide OPBP-1-loaded trimethyl chitosan hydrogel for cancer immunotherapy, J. Control. Release. 334 (2021) 376–388. https://doi.org/10.1016/j.jconrel.2021.04.036.

[39] J. Fetse, Z. Zhao, H. Liu, U.-F. Mamani, B. Mustafa, P. Adhikary, M. Ibrahim, Y. Liu, P. Patel, M. Nakhjiri, M. Alahmari, G. Li, K. Cheng, Discovery of Cyclic Peptide Inhibitors Targeting PD-L1 for Cancer Immunotherapy, J. Med. Chem. 65 (2022) 12002–12013. https://doi.org/10.1021/acs.jmedchem.2c00539.

[40] S. Ishiguro, D. Upreti, M. Bassette, E.R.A. Singam, R. Thakkar, M. Loyd, M. Inui, J. Comer, M. Tamura, Local immune checkpoint blockade therapy by an adenovirus encoding a novel PD-L1 inhibitory peptide inhibits the growth of colon carcinoma in immunocompetent mice, Transl. Oncol. 16 (2022) 101337. https://doi.org/10.1016/j.tranon.2021.101337.

[41] M. Zhang, Z. Fang, H. Zhang, M. Cui, M. Wang, K. Liu, Reversing tumor immunosuppressive microenvironment via targeting codelivery of CpG ODNs/PD-L1 peptide antagonists to enhance the immune checkpoint blockade-based anti-tumor effect, Eur. J. Pharm. Sci. 168 (2022) 106044. https://doi.org/10.1016/j.ejps.2021.106044.

[42] Z. Zhou, X. Li, G. Yang, J. Wang, B. Li, Y. Huang, J. Yan, K. Tao, Targeting β-catenin and PD-L1 simultaneously by a racemic supramolecular peptide for the potent immunotherapy of hepatocellular carcinoma, Theranostics. 13 (2023) 3371–3386. https://doi.org/10.7150/thno.83377.

[43] Z.-Z. Yu, Y.-Y. Liu, W. Zhu, D. Xiao, W. Huang, S.-S. Lu, H. Yi, T. Zeng, X.-P. Feng, L. Yuan, J.-Y. Qiu, D. Wu, Q. Wen, J.-H. Zhou, W. Zhuang, Z.-Q. Xiao, ANXA1-derived peptide for targeting PD-L1 degradation inhibits tumor immune evasion in multiple cancers, J. Immunother. Cancer. 11 (2023) e006345. https://doi.org/10.1136/jitc-2022-006345.

[44] W. Ji, B. Zhang, X. Sun, L. Ma, X. Zhang, W. Qian, W. Fu, J. Li, D. Zhu, Inhibition of tumor immune escape by blocking PD-1/PD-L1 engagement with dual-targeting molecularly imprinted polymer layer, Cancer Nanotechnol. 14 (2023) 51. https://doi.org/10.1186/s12645-023-00209-3.

[45] S. Song, M.K. Shim, S. Yang, J. Lee, W.S. Yun, H. Cho, Y. Moon, J.Y. Min, E.H. Han, H.Y. Yoon, K. Kim, All-in-one glycol chitosan nanoparticles for co-delivery of doxorubicin and anti-PD-L1 peptide in cancer immunotherapy, Bioact. Mater. 28 (2023) 358–375. https://doi.org/10.1016/j.bioactmat.2023.05.016.

[46] W. Shen, P. Shi, Q. Dong, X. Zhou, C. Chen, X. Sui, W. Tian, X. Zhu, X. Wang, S. Jin, Y. Wu, G. Chen, L. Qiu, W. Zhai, Y. Gao, Discovery of a novel dual-targeting D-peptide to block CD24/Siglec-10 and PD-1/PD-L1 interaction and synergize with radiotherapy for cancer immunotherapy, J. Immunother. Cancer. 11 (2023) e007068. https://doi.org/10.1136/jitc-2023-007068.

[47] M. Rui, W. Zhang, K. Mi, H. Ni, W. Ji, X. Yu, J. Qin, C. Feng, Design and evaluation of α-helix-based peptide inhibitors for blocking PD-1/PD-L1 interaction, Int. J. Biol. Macromol. 253 (2023) 126811. https://doi.org/10.1016/j.ijbiomac.2023.126811.
